# A New Dolphin Species, the Burrunan Dolphin *Tursiops australis* sp. nov., Endemic to Southern Australian Coastal Waters

**DOI:** 10.1371/journal.pone.0024047

**Published:** 2011-09-14

**Authors:** Kate Charlton-Robb, Lisa-ann Gershwin, Ross Thompson, Jeremy Austin, Kylie Owen, Stephen McKechnie

**Affiliations:** 1 Centre for Environmental Stress and Adaptation Research, School of Biological Sciences, Monash University, Clayton, Victoria, Australia; 2 Australian Centre for Biodiversity, School of Biological Sciences, Monash University, Clayton, Victoria, Australia; 3 Tasmanian Museum and Art Gallery, Hobart, Tasmania, Australia; 4 South Australian Museum, Adelaide, South Australia, Australia; 5 Australian Centre for Ancient DNA, University of Adelaide, Adelaide, South Australia, Australia; 6 Sciences Department, Museum Victoria, Carlton Gardens, Melbourne, Victoria, Australia; Smithsonian Institution National Zoological Park, United States of America

## Abstract

Small coastal dolphins endemic to south-eastern Australia have variously been assigned to described species *Tursiops truncatus*, *T. aduncus* or *T. maugeanus*; however the specific affinities of these animals is controversial and have recently been questioned. Historically ‘the southern Australian *Tursiops*’ was identified as unique and was formally named *Tursiops maugeanus* but was later synonymised with *T. truncatus*. Morphologically, these coastal dolphins share some characters with both aforementioned recognised *Tursiops* species, but they also possess unique characters not found in either. Recent mtDNA and microsatellite genetic evidence indicates deep evolutionary divergence between this dolphin and the two currently recognised *Tursiops* species. However, in accordance with the recommendations of the Workshop on Cetacean Systematics, and the Unified Species Concept the use of molecular evidence alone is inadequate for describing new species. Here we describe the macro-morphological, colouration and cranial characters of these animals, assess the available and new genetic data, and conclude that multiple lines of evidence clearly indicate a new species of dolphin. We demonstrate that the syntype material of *T. maugeanus* comprises two different species, one of which is the historical ‘southern form of *Tursiops*’ most similar to *T. truncatus*, and the other is representative of the new species and requires formal classification. These dolphins are here described as *Tursiops australis* sp. nov., with the common name of ‘Burrunan Dolphin’ following Australian aboriginal narrative. The recognition of *T. australis* sp. nov. is particularly significant given the endemism of this new species to a small geographic region of southern and south-eastern Australia, where only two small resident populations in close proximity to a major urban and agricultural centre are known, giving them a high conservation value and making them susceptible to numerous anthropogenic threats.

## Introduction

Delphinids are the most ecologically diverse cetacean, occurring across a range of latitudes, in coastal and oceanic waters, and in estuarine and freshwater habitats [1]. In the last 25 years molecular techniques have markedly improved our understanding of cetacean taxonomy, including recognition of undescribed taxa within family Delphinidae [2], [3]. However, relationships within sub-family Delphininae remain uncertain [4], [5], [6], largely due to their rapid global radiation and the ability of species to locally adapt [4]. Several species are distributed globally but show fine scale local population structure [7].

The genus *Tursiops* has been plagued with controversy with historically upwards of 20 species described, all synonymised with *T. truncatus* [8]. Only recently *T. aduncus* has been revalidated as the second *Tursiops* species, this based on morphological and mitochondrial DNA data [5], [9], [10], [11]. In fact numerous studies have demonstrated that *Tursiops* is polyphyletic [5], [12], [13], [14], [15]. However, there is still controversy with two new distinct *Tursiops* species recently suggested [12], [13], [14]. In Australia, all *Tursiops* species have been historically recognised as *T. truncatus* [16]. However, Möller and Beheregaray [17] genetically confirmed the presence of *T. aduncus* off eastern Australia, while in Western Australia *aduncus* and *truncatus*-type haplotypes are also present [18].

In south-eastern Australia, morphological variation within *Tursiops* has been described for several decades [16], [19], [20]. In 1919, Scott and Lord [19] detailed the external and skeletal morphology of a unique, sexually dimorphic, southern form of *Tursiops* (as *T. tursio*). A single male specimen was captured by H.H. Scott in 1902 in the Cataract Gorge, Launceston, Tasmania. At the time, media reports, exhibition signage, and Scott's own handwritten notes (held in the Queen Victoria Museum and Art Gallery) indicated that he believed it belonged to a distinct southern form of *T. tursio*. In 1914, a female specimen was obtained in the North Esk, Launceston, Tasmania. As stated, Scott and Lord [19] believed that the male and female belonged to the same species, and accounted for their numerous morphological differences by adding sexual dimorphism to the list of characters separating this southern form from the northern form of *Tursiops*. Iredale and Troughton [21] formally named Scott and Lord's form [19], *Tursiops maugeanus*. Validity of the species has not been accepted by later authors and has been synonymised with *T. truncatus* [20], [22]. In addition, the whereabouts of the *T. maugeanus* ‘holotype’ has been listed as unknown [23]. We have recently located the male and female syntypes of *T. maugeanus* and incorporated them into this contemporary analysis.

In the current study, morphology indicates two forms of ‘bottlenose’ dolphin in south-eastern Australia, a physically smaller coastal form in semi-enclosed water bodies, and a larger more robust ‘offshore’ form. Locations of beach-cast dolphins suggest these two forms are parapatric, at least across some of their range. The smaller coastal form has been noted as both *T. truncatus* and *T. aduncus* [24], [25] and due to the historical and current ambiguity of species identification, this form has more recently been referred to as *Tursiops* sp., southern Australian bottlenose dolphin (SABD) [13], [14], [26], [27].

Charlton et al. [13] using the mtDNA control region first highlighted the divergent mtDNA lineage of SABD (using samples from the two Victorian populations), showing they did not cluster with *Tursiops*, *Delphinus* or *Stenella* species found world-wide. The average sequences divergence of the Victorian SABD to *T. truncatus* and *T. aduncus* (5.5% and 9.1% respectively [13]) was greater than that observed between recognised species within each of the *Cephalorhynchus* (2.5–4%) and *Lagenorhynchus* (4.5–6.4%) genera [28]. Charlton et al. [13] concluding that these populations may represent an undescribed taxon. Möller et al. [14] later provided evidence for three genealogically distinct, reciprocally monophyletic, mtDNA lineages among the dolphins in southern Australia. Complementary microsatellite data indicated reproductive isolation among lineages [14]. Two of these lineages corresponded to published sequences of *T. truncatus* and *T. aduncus* [14]. The third lineage, including all SABD animals, was novel, the data suggesting it is a sister taxon of *Lagenodelphis hosei* (Fraser's Dolphin). Kingston et al. [4] using mtDNA control region haplotypes from Charlton et al. [13] confirmed SABD as a monophyletic clade separate from *Tursiops* species, but with the sister taxa *Sousa chinensis* (Indo-Pacific Humpback Dolphin). Unlike other recently recognised species, *Orcaella heinsohni* [3] and *Sotalia guianensis* [2], where the classification within pre-existing genera was clear, SABD does not associate unambiguously with any described genus, and particularly not with either recognised species of ‘bottlenose dolphin’ in the genus *Tursiops*.

In 2004 a specialized Workshop on Cetacean Systematics was held to review cetacean taxonomy and provided criteria for species delimitation [29]. At that workshop it was agreed that multiple lines of evidence are required to demonstrate “irreversible divergence” with criteria from both morphological and genetic data taken as proxies for reproductive isolation. The “ideal data set” will include both morphological data and data from multiple genetic loci [29].

Currently there are numerous Species Concepts, each with underlying properties that represent thresholds crossed by diverging lineages, different subgroups of biologist advocate different species concepts but they all exhibit underlying conceptual unity [30]. De Queiroz [30] highlights that unity and proposes a Unified Species Concept, stating that species are separately evolving *metapopulation* lineages on different evolutionary trajectories, the farther along process of divergence, the larger the number of differences. In this Unified Species Concept, any property (line of evidence) that provides evidence of lineage separation is relevant in species delimitations including genetic, morphological, ecological or behavioural [30]. A highly corroborated hypothesis of the existence of a new species requires multiple lines of evidence, the farther along the process of divergence, the easier it becomes to find and highlight evidence of separation. The Unified Species Concept [30] was used in the more recent 2009 Workshop for Defining Subspecies: Developing Guidelines for Marine Mammals [31] and whilst this Workshop was specific to the lower end continuum of subspecies differentiation, the Concept was used to highlight the “*differentiation that characterizes the process of speciation*” [31].

Since 2003 we have carried out extensive surveys, sampling and characterisation of ‘bottlenose’ dolphins from Victorian and Tasmanian coastal waters, using museum specimens, beach-cast strandings, live sightings and biopsies. In light of the confusion surrounding the taxonomy of these animals we use existing [13], [14] and new genetic data, external and cranial morphometrics, incorporating the syntypes of *T. maugeanus*, and assess the taxonomic status of these animals. Consistent with the recommendations of the Workshop on Cetacean Systematics [29], and the Unified Species Concept [30] these multiple lines of evidence are used to establish the SABD as a new species of dolphin.

## Methods

### Study location

South-eastern Australia, encompassing coastal waters of Victoria and Tasmania (Figure S1). Southern Queensland, Australia (Museum specimens only).

### Cranial morphology

Forty commonly used cranial measurements and tooth counts were taken from 44 specimens of ‘bottlenose’ dolphins from across Australia (Table 1 & 2; Table S1). Only adult specimens with complete data sets were used (those exhibiting secure fusion between maxillae and cranium [16]). All measurements were taken by the first author. Specimens were collected from locations across coastal Victoria (Museum Victoria (MV) (n = 26); and Monash University (MU) (n = 5) collections), Tasmania (Tasmanian Museum and Art Gallery (TMAG) (n = 5); Queen Victoria Museum and Art Gallery (QVMAG) (n = 5) collections) and Queensland Museum (QM) (n = 4) (Table 1). Cranial measures largely followed Kemper [20] and Wang et al. [32]. We included an undescribed measure, anterior pterygoid apex to palatine (APAP); plus two undescribed qualitative features, shape of the palatine and flattening on the maxilla at the base of the rostrum (Table S1).

**Table 1:** List of all ‘bottlenose’ dolphin specimens examined with collection information.

Museum -	Collection	MU Ext. Morph.	Date	Sex	Collection location	
University code		Label				
C29579	MV	-	14/10/1985	F	Western Beach	PPB
C29587	MV	-	1/06/1992	M	Kennedy's Point	WPB
C29667	MV	-	8/01/1987	F	Ocean Grove	Vic
C24944	MV	-	2/06/1967	F	Elwood	PPB
C28760	MV	-	13/11/1992	-	Sandringham	PPB
C29580	MV	-	17/01/1986	M	Murrells Beach	Vic
C29577	MV	-	23/07/1985	F	Safety Beach	PPB
C29586	MV	-	27/07/1991	M	Rippleside	PPB
C10357	MV	-	-	-	-	
C31642	MV	-	-	-	-	
C35986	MV	-	4/04/2006	F	Mitchell River	Gips
C35987	MV	-	21/07/2006	M	Hollands Landing	Gips
C25071	MV	-	-	-	Stingaree Beach	PPB
Unknown	MV	-	-	-	-	
C29506	MV	-	16/04/1994	F	Sorrento	PPB
C11271	MV	-	-	-	-	
1365*	**QVMAG**	**-**	**11/11/1914**	**F**	**North Esk River**	**Tas**
A1759	TMAG	-	21/02/2003	-	Marion Bay	Tas
A2430	TMAG	-	-	-	-	
1946/7	QVMAG	-	16/01/1947	M	North Esk River	Tas
1972/1/35	QVMAG	-	1965		Bass Strait	Tas
1360**	QVMAG	-	1902	M	Cataract Gorge	Tas
C31643	MV	-	-	F	-	
A2425	TMAG	-	-	-	-	
A198	TMAG	-	1919	-	East Coast	Tas
C24987	MV	-	18/05/1967	-	Lorne	Vic
C29585	MV	-	13/05/1990	M	Wild Dog Creek	Vic
C29581	MV	-	22/01/1986	M	Port Fairy	Vic
TMAG unreg	TMAG	-	2007	-	-	
WAPSTRA	QVMAG	-	10/02/1981	-	Eaglehawk Neck	Tas
MU270508	MU		27/05/2008	F	Cape Conran	Vic
C35965	MV	MU141206a	14/12/2006	M	Lake Wellington	Gips
C35985	MV	MU011206	1/12/2006	M	Blonde Bay	Gips
C35966	MV	MU141206b	14/12/2006	M	Lake Wellington	Gips
C36750	MV	MU041107	4/11/2007	M	Paynesville	Gips
C35969	MV	MU080306	8/03/2006	M	Phillip Island	Vic
C35968	MV	MU251007	25/10/2007	M	Tucker Point	Gips
MU210108	MU	MU210108	21/01/2008	M	Beaumaris	PPB
MU230108	MU	MU230108	23/01/2008	M	Point Henry	PPB
MU230607	MU	MU230607	23/06/2007	F	Point Ricardo	Vic
MU220108	MU	MU220108	22/01/2008	F	Killarny	Vic
-	-	MU021108	2/11/2008	M	Swan Reach	Gips
-	-	MU291007	29/10/2007	F	Jones Bay	Gips
-	-	MU230407	23/04/2007	M	San Remo	WPB
-	-	MU190905	19/09/2005	F	Corio Bay	PPB
-	-	MU271006	27/10/2006	M	Port Fairy	Vic
-	-	MU280405	28/04/2005	F	Kennett River	Vic
-	-	MU010709	1/07/2009	M	Portland	Vic
JM1230	QM	-	6/02/1976	-	Moreton Bay	Qld
JM11375	QM	-	4/03/1996	M	Bargara Beach	Qld
5241	QM	-	1983	-	Nth Stradbroke Is	Qld
6428	QM	-	22/02/1987	M	Yellow Patch	Qld
4155	QM	-	-	-	Townsville	Qld

**MV** Museum Victoria; **MU** Monash University; **QVMAG** Queen Victoria Museum and Art Gallery; **TMAG** Tasmania Museum and Art Gallery; **QM** Queensland Museum:**Tas** Tasmanian waters; **Vic** Victorian coastal water; **PPB** Port Phillip Bay, Victoria; **WPB** Westernport Bay, Victoria; **Gips** Gippsland Lakes, Victoria; **QLD** Queensland waters.

**Table 2:** Basic cranial measures statistics for *Tursiops australis* sp. nov., *Tursiops truncatus* and *Tursiops aduncus*.

		*Tursiops australis*				*Tursiops truncatus*				*Tursiops aduncus*			
Measure	*n*	Mean (mm)	Range (mm)		*n*	Mean (mm)	Range (mm)		*n*	Mean (mm)	Range (mm)		significance
			min	max			min	max			min	max	
BL	23	35.63	33.43	38.28	8	37.41	34.92	38.91	2	33.70	32.64	34.77	ns
CBL	27	493.58	470	513	13	527.88	505.5	547	5	441.00	424	455	**
DFWM	27	18.22	9.29	30.14	13	16.10	8.22	27.37	5	19.44	17.61	21.96	ns
DFWN	27	12.50	5.05	17.53	13	16.02	7.2	23.26	5	12.58	9.71	16.22	*
GLPT	27	60.12	53.74	66.69	13	75.89	67.16	82.24	3	63.71	61.49	66.88	**
GLPTF	27	114.59	102.83	155.71	13	115.85	108.8	125.62	5	95.69	85.74	105.46	ns
GWPTF	27	82.96	78.6	87.87	13	84.28	76.97	89.88	5	76.86	72.85	81.39	ns
GWEN	27	60.42	55.3	64.97	13	58.95	54.23	64.05	5	50.05	47.91	55.01	ns
GWIN	27	66.98	58.79	74.94	13	76.64	70.44	84.74	5	57.59	54.34	60.69	**
GPRW	27	213.35	198	231.5	13	237.62	224	251	5	193.90	180	200.50	**
GPOW	27	238.93	222	255.5	13	268.58	253.5	286.5	5	213.00	198	224	**
GWPX	27	94.72	85.55	103.49	13	97.98	89.12	110.62	5	77.46	73.03	83.13	ns
GPARW	27	185.45	174.58	194.83	13	190.16	181.62	196.31	-	-	-	-	-
LAL	27	52.03	44.79	59.64	13	62.23	54.14	70	5	42.68	39.48	46.45	**
LO	27	69.95	63.29	77.22	13	69.06	61.01	77.16	5	60.63	54.60	64.80	ns
LTRL	27	232.87	219	252.5	13	247.83	229.5	264	5	219.90	213.50	227	**
LWPTF	27	162.64	145.13	175.66	13	155.36	135.78	167.93	5	148.85	141	161.58	*
MFL	27	142.19	133.56	153.92	13	149.54	132.16	163.94	5	119.90	109.79	127.41	*
MH	27	91.78	87.28	97.06	13	97.48	89.15	105.05	5	80.25	77.75	83.48	**
ML	27	423.30	405	441	13	457.46	433	474	5	373.20	360.50	384.50	**
MSL	27	66.65	58.57	74.12	13	73.02	51.06	87.76	5	63.16	56.85	68.73	*
POL	22	34.20	25.13	38.09	8	34.04	31.8	36.29	2	30.83	29.90	31.77	ns
PRW	27	48.49	39.74	58.7	13	50.20	42.35	58.23	5	33.31	30.93	35	ns
RL	27	280.37	265.5	295	13	303.69	291.5	326	5	254.30	243	264	**
RWB	27	132.58	123.46	145.29	13	143.05	136.29	158.89	5	103.38	93.78	107.78	**
RW60	27	93.58	81.2	120.6	13	106.46	97.88	117.62	5	77.90	71.99	82.72	**
RWM	27	79.44	70.74	87.55	13	88.84	79.24	100.82	5	62.64	60.09	65.44	**
RW75	27	63.48	55.02	71.7	13	70.97	57.67	82.73	5	50.05	46.82	53.76	**
TREN	27	327.98	307.5	348	13	353.23	337	375.5	5	295.20	290	305	**
TRIN	27	333.43	318	354	13	360.58	339	375	3	297.17	292.50	305	**
UTLTR	27	236.59	223	250	13	253.04	240	267	5	209.10	200	216	**
VW	27	43.15	36.05	49.74	13	49.96	35.81	63.52	5	30.52	23.05	36.22	**
ZW	27	228.52	209	242.5	13	263.27	246	278	5	204.50	191	221.50	**
APAP	27	55.98	45.27	63.92	13	42.18	23.88	57.84	5	41.42	31.36	47.13	**
TPC	27	161.01	140.89	178.56	12	176.74	147.05	206.74	5	159.02	151.84	169.63	**
WAS	27	73.41	64.87	78.12	13	85.48	79.24	97.63	5	62.42	55.85	67.07	**
**Tooth counts**													
TTLL	25	22.84	21	26	11	22.09	20	24	5	24.20	22	27	ns
TTLR	26	23.12	21	26	11	22.18	20	25	5	24.20	23	26	ns
TTUL	25	23.88	22	27	11	23.36	20	26	5	23.20	21	25	ns
TTUR	26	23.85	22	28	11	23.27	20	26	5	23.00	21	25	ns

*significant (p<0.05);

**highly significant (p<0.001); ns not significant.

As historically all *Tursiops* species were recognised as *T. truncatus*, QM specimens remain listed as either *Tursiops* sp. or *T. truncatus*. However *T. aduncus* is now known to be present in Queensland waters [33]. As such, we enlisted the technique used by Perrin et al. [34] in their assessment of the holotype specimen of *T. aduncus*, and used the range (min-max) of cranial measures presented in Wang et al [32], to conclude the four QM specimens were referable of *T. aduncus*.

### External morphology

Eighteen external morphometrics (Table 3; Table S2) were taken from 17 ‘bottlenose’ dolphins from coastal Victoria (Table 1). Beach-cast dolphins were opportunistically measured during 2005–2009, by the first author and researchers at the Dolphin Research Institute, Monash University and the Department of Sustainability and Environment (Victorian Government). Animals were excluded from analysis if data was incomplete, bloating due to decomposition had occurred, or if the animal was a juvenile (less than 220 cm in length).

**Table 3:** Basic external measures statistics for *Tursiops australis* sp. nov. and *Tursiops truncatus* from Victoria waters, south-eastern Australia.

		*Tursiops australis*			*Tursiops truncatus*		
Measure	*n*	Mean (cm)	Range (cm)	*n*	Mean (cm)	Range (cm)	significance
UJAM	9	10.8	9.4–12	4	11.6	11–12.5	NA
UJGAP	12	28.7	24–30.5	5	33.2	32–35	**
UJEYE	12	35.4	30.5–39	5	38.7	34–40.5	*
UJBH	12	37.1	33.5–40	5	39.9	37–43	*
UJDF	9	113.8	101–119	3	130	125–135	NA
UJTDF	12	156.8	143–168	5	175.3	172–183	**
TLEN	12	257.1	235.5–278	5	295	283–302	**
UJFLIP	12	56.9	50.5–61	5	61.7	60–63	*
UJGEN	9	157.4	146–192	4	195.4	180–20	NA
UJANU	12	181.0	166–194	5	208.6	205–212	**
LFLIP	12	46.0	36–48.5	5	44.9	42.5–47	ns
WFLIP	12	17.8	13–26	5	16.1	15–18	ns
WFLU	12	62.9	56.5–68	5	68.3	64.5–78	*
DCN	9	4.2	2–5.5	4	5	3.2–8	NA
HD	12	26.3	24–28	5	29	23.5–34	*
PROJ	9	1.0	0.5–2	4	1.3	0.5–2	NA
GIRMAX	11	144.6	112–164	5	138.2	123–154	NA
GIRANU	11	75.3	68–82	3	79	71–84	NA

NA measure eliminated from study;

*significant (p<0.05);

**highly significant (p<0.001); ns not significant.

### Morphological data analysis

Multivariate analyses of variance was used to test for sexual dimorphism in both cranial and external morphometrics datasets (not found, therefore males and females were pooled in further analyses) (cranial MANOVA, F_64,18_ = 1.289, p = 0.28, male = 17 female = 9; external MANOVA, F_11,6_ = 1.056, p = 0.449, male = 11 female = 6). Data were standardized by converting the raw data to z-scores. Hierarchical cluster analysis (HCA) using Euclidean pair-group average, discriminant function analyses (DFA) and principal component analyse ((PCA) presented as supporting information only Figure S2 & S3; Table S3 & S4) were used to determine whether specimens formed distinct morphological groups or ‘clusters’. Genotypes of specimens were overlain to assess if assignment of clusters from HCA and DFA were indicative of the ‘species’ mtDNA haplotypes and if there was appropriate assigment into species ‘type’. DFA was also used to identify the measures which drove separation of clusters. MANOVA was used to test whether measures were statistically significant between clusters. All analyses were completed using Systat v13 [35] and Past v1.94b [36].

### Mitochondrial DNA sequencing

Skin samples were collected from beach-cast dead ‘bottlenose’ dolphins from coastal Victoria and stored in saline solution of 20% dimethyl-sulfoxide (DMSO), 0.25 M EDTA, saturated with NaCl, pH 7.5 [37]. Where skin samples were not available (museum specimens) tooth samples were collected. Numerous biopsy samples were taken from free-ranging dolphins during 2006–2008 using the PAXARMS biopsy system [38]. Several biopsy samples were used to verify genetic ‘type’ from the living populations (data not presented here).

Tooth samples were individually stored in sterilized Falcon tubes. Each tooth was sectioned and decontaminated by being submerged for 10 min in 12% sodium hypochloride [39]. Sections were decalcified for up to four days using Morse's Solution (10% Sodium Citrate, 20% Formic Acid) until ‘rubbery’ and flexible. Morse's Solution was used as it does not degrade DNA quality [40]. Tooth samples were run from two separate extractions and two separate PCR reactions, including negative controls.

Total genomic DNA was extracted from skin and tooth samples using a Puregene Tissue kit (Gentra Systems) following manufacturer's instructions, with modification for the teeth. Samples were analysed for quality and quantity of genomic DNA using a NanoDrop ND-1000 Spectrophotometer. PCR amplification and sequencing of a ∼450-bp fragment of the mtDNA control region and ∼1200-bp of cytochrome *b* was undertaken following modified methods outlined in Charlton et al. [13] and Möller et al. [14] respectively. Tooth sample modifications include 1) an additional 4 µl of proteinase K during extraction (following manufacturer's instructions) 2) use of 6 µl of 30 ng/µl gDNA in the PCR reaction due to reduction of quality and quantity of DNA compared to skin samples and 3) use of Bio-X-Act Short for all samples (Bioline). All PCR products were sent to Macrogen, Korea, for purification and sequencing. Capillary electrophoresis (CE) was conducted on an Applied Biosystems ABI 3730xl DNA analyzer. Purified PCR products from tooth samples were also sequenced at Micromon, Monash University, with CE on an Applied Biosystems 3730S Genetic Analyser.

### Syntype specimens of Tursiops maugeanus

Small bone samples were taken from both syntype specimens of *Tursiops maugeanus* [19], [21] QVMAG#1365 and QVMAG#1360 using pre-sterilised 5 mm drill bits and a slow-speed hand drill. All pre-PCR work was conducted at a dedicated ancient DNA facility (Australian Centre for Ancient DNA, University of Adelaide, South Australia) using stringent ancient DNA precautions and controls [41]. DNA was extracted from 100 mg bone powder using a modified Qiagen DNeasy Blood and Tissue kit [42]. A negative extraction control was included to monitor contamination during the extraction process. PCR amplification and sequencing of a 1124-bp region of the cytochrome *b* gene and 417-bp region of the control region was carried out using seven and three sets of primers respectively, each amplified a 132–200-bp overlapping fragment (Table S5). PCR amplifications were carried out in 25 ul reaction volumes containing, 2 mM MgSO4, 0.25 mM each dNTP, 1× PCR buffer (Invitrogen), 0.4 uM of each primer, 1 mg/ml RSA (Sigma) and 0.5 U of Platinum Taq Hifidelity (Invitrogen). PCR amplification was performed under the following conditions: 94°C 1 min, then 50 cycles of 94°C 15 s; annealing 55°C 15 s; 68°C 30 s, followed by a final elongation step of 68°C for 10 min. A PCR negative control and negative extraction control were included in all amplification attempts. PCR products were purified with Ampure (Agencourt) according to manufacturer's instruction and Sanger sequencing was undertaken using the ABI prism Big Dye Terminator Cycle sequencing kit (PE Applied Biosystems, Foster City, CA). CE was carried out on an ABI 3130XL DNA analyser and raw sequences were edited using Sequencher (Genecodes). To ensure authenticity and reliability of the sequence obtained from the historical specimens, all PCR and sequencing was repeated providing independent and duplicate coverage of all sequenced bases.

### Mitochondrial DNA sequence data analysis

All sequences were edited and aligned by eye using Mega5 [43]. Each individual sequence was assigned to a haplotype. These haplotypes were used to confirm the genetic identity of the specimens represented in the morphological data and were also compared to the available sequences of both *Tursiops truncatus* and *T. aduncus*.

Whilst the mtDNA analyses was conducted on both cytochrome *b* and control region in the current study, in order to assess the phylogenetic affinities of these animals, comparisons must be made with additional taxa in the subfamily Delphininae. In order to achieve this we have chosen to use the mtDNA control region in isolation, as conducting a consolidation analyses using both mtDNA regions of the wider Delphininae taxa would involve taking individual haplotypes from GenBank and assuming individual identity and locale, and thus may misrepresent the affinities of each taxa. Mitochondrial DNA control region sequences representing multiple genera within subfamily Delphininae were downloaded from Genbank, including those previously reported in Charlton et al. [13] as AustVic, representing additional Victorian SABD haplotypes, and the *T. aduncus* holotype sequence (Genbank accession #DQ517442; Museum accession #ZMB66400). A model and parameters for the phylogenetic reconstruction were determined empirically using likelihood via Mega5 [43]. The Bayesian Information Criterion scores (BIC) and Akaike Information Criterion, corrected (AIC) indicated Hasegawa-Kishino-Yano model [44] plus Gamma, with alpha (gamma, K = 5) = 0.3183 distribution, was the most appropriate model given the above data. The dataset was analysed using Maximum Likelihood (ML), Neighbour-Joining (NJ) and Bayesian inference. The ML analysis was conducted in Mega5 [43] and the NJ analysis was conducted in PAUP v4.0b10 [45] using the above model. Reliability of the nodes for all trees was assessed using 1,000 bootstrap replicates. Bayesian phylogenetic inference was conducted through Mr Bayes 3.1.2 [46]. The Monte Carlo Markov Chain (MCMC) was run over 10,000,000 iterations, with a sampling frequency of 1,000. All other parameters in Mr Bayes were set to default. The analysis was run over 2 replicates to assure convergence on a topology. The *Lagenorhynchus acutus* sequence was used as outgroup (see Table S6 for Genbank accession numbers).

### Animal Ethics and Research Permit approval

Collection of samples was approved by Monash University Biological Sciences Animal Ethics Committee (AEC approval BSCI# BSCI/2008/21) and Victorian State Government, Department of Sustainability and Environment (DSE) Wildlife Act 1975 Research Permit (Permit No: 10005013).

## Results

### Cranial morphology

**Figure 1:**
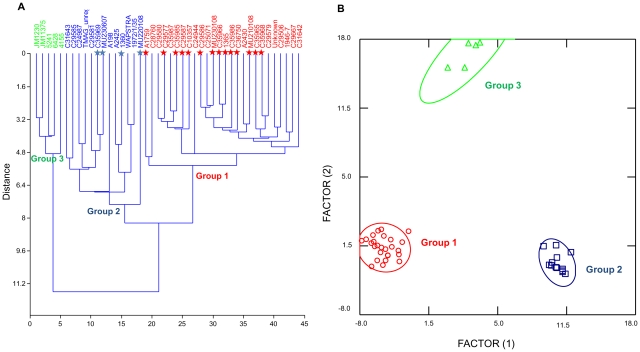
Graphic analyses on cranial morphology delineating *Tursiops australis* sp. nov. and *Tursiops* species (A–B). Red = Group 1: *Tursiops australis* sp. nov., blue = Group 2: *T. truncatus*, green = Group 3: *T. aduncus*. Individuals with known mtDNA sequence are indicated by★with the appropriate species colour code. (**A**) Hierarchical multivariate cluster analysis on cranial morphological traits showing three highly supported groups (cophenetic correlation 0.8337). *Tursiops australis* sp. nov. holotype (QVMAG#1365) in Group 1, and *Tursiops maugeanus* male (QVMAG#1360) in Group 2. (**B**) Discriminant function analyses scatterplot of canonical scores on cranial morphological traits delineating *Tursiops australis* sp. nov., *T. truncatus* and *T. aduncus*.

Hierarchical cluster analysis was performed on all cranial variables for 44 specimens and showed three highly supported groups (cophenetic correlation of 0.8337) (Figure 1a). Group 1 was largely represented by specimens collected in enclosed coastal waters of Victoria, Group 2 was largely represented by specimens collected from ‘offshore’ coastal waters of Victoria and Tasmania, whilst Group 3 was represented only by Queensland specimens.

Discriminant function analysis (DFA) was used to determine whether cranial characteristics would distinguish the same groupings identified by the cluster analysis. The DFA scatterplot of canonical scores clearly show the three well separated ‘groups’ (Figure 1b). *A posteriori* classifications were 100% correct. Canonical Discriminant function weighting identified rostrum measures RWM and RW60, width measure GPOW (greatest postorbital width) and length measure TRIN (tip of the rostrum to internal nares) as important characters defining the groups (Table S7). Thirty-two of the 36 skull measures differed significantly between the ‘groups’ (Wilks' λ MANOVA, F_66,18_ = 12.839, p<0.001) (Table 2).

### External morphology

**Figure 2:**
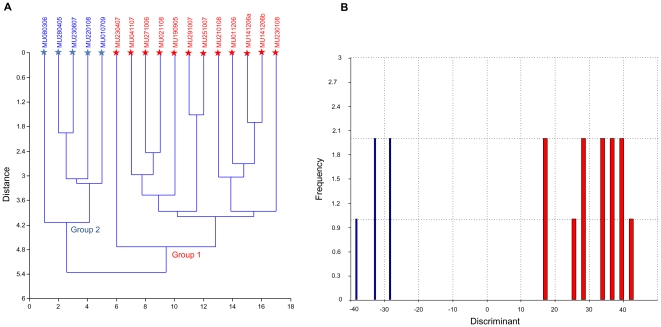
Graphic analyses on external morphology delineating *Tursiops australis* sp. nov. and *Tursiops truncatus* (A–B). Red = Group 1: *Tursiops australis* sp. nov., blue = Group 2: *T. truncatus*. Individuals with known mtDNA sequence are indicated by★with the appropriate species colour code. (**A**) Hierarchical multivariate cluster analysis on external morphological traits showing two highly supported groups (cophenetic correlation of 0.747). (**B**) Discriminant function analyses on external morphological traits delineating *Tursiops australis* sp. nov. and *T. truncatus* (Hotellings t2: p = 0.0224).

Hierarchical cluster analyses performed on 14 variables using 17 specimens clearly showed two highly supported groups (cophenetic correlation of 0.747) (Figure 2a).

DFA was also used on external characters to ascertain whether specimens were classified into the same groups as the cranial analyses. The histogram of dolphin specimens along the discriminant axis clearly show the two ‘groups’ well separated (Hotellings t2 p = 0.022) (Figure 2b). Again, *a posteriori* individual group assignments corresponded exactly. Discriminant function weighting showed several length measures (UJBH, UJEYE, UJGAP) and width of flukes (WFLU) as important characters defining the groups (Table S8). Nine measures of the 14 measures differed significantly between the ‘groups’ (Wilks’ λ MANOVA, F_11,2_ = 64.32, p<0.001) (Table 3).

### Molecular analyses: Mitochondrial DNA sequence data

Molecular analyses were limited to samples included in the cranial (n = 18) and external (n = 17) morphology analyses from south-east Australian samples (ten of the animals were represented in both cranial and external datasets). DNA data could not be obtained from QM skulls. From the 25 samples where DNA was available no *T. aduncus* mtDNA types were found.

### Cytochrome b

A 1086-base sequence of the mtDNA cytochrome *b* region was obtained from 18 samples representing both cranial and external morphology groups (Table S9). Six unique haplotypes were identified, three representing *T. australis* sp. nov. and three representing *T. truncatus*, defined by 62 variable sites (59 transition substitutions, 3 transversion substitutions). Forty-eight fixed site differences were noted between the two species (Table 4). *Tursiops australis* sp. nov. showed minimal intra-specific variation, with three variable sites, whilst *T. truncatus* showed more variation with 12 variable sites.

**Table 4:** Mitochondrial DNA cytochrome *b* region diagnostic sites.

										1	1	1	2	2	2	2	2	3	3	4	4	4	4	4	4	5	5	5	5	5	5
	1	2	2	3	3	4	5	7	9	2	3	5	1	2	4	7	8	1	2	1	4	5	5	8	9	0	2	2	2	4	6
	9	2	8	4	7	6	5	9	7	1	9	4	5	0	1	5	6	9	6	9	9	4	7	4	4	5	3	6	9	7	3
***Tursiops australis*** **p sp. nov.**																															
**Burru Cytb1 holotype**	**C**	**A**	**T**	**G**	**T**	**C**	**T**	**G**	**T**	**T**	**A**	**C**	**T**	**T**	**C**	**C**	**C**	**C**	**C**	**C**	**C**	**T**	**A**	**T**	**T**	**C**	**C**	**C**	**G**	**A**	**T**
Burru Cytb1	.	.	.	.	.	.	.	.	.	.	.	.	.	.	.	.	.	.	.	.	.	.	.	.	.	.	.	.	.	.	.
Burru Cytb3	.	.	.	.	.	.	.	.	C	.	.	.	.	.	.	.	.	.	.	.	.	.	.	.	.	.	.	.	.	.	.
Burru Cytb4	.	.	.	.	.	.	.	.	.	.	.	.	.	.	.	.	.	.	.	.	.	.	.	.	.	.	.	.	.	.	.
***Tursiops truncatus***																															
***T. maugeanus*** ** TTCytb5 lectotype**	**T**	**G**	**C**	**A**	**C**	**T**	**C**	**A**	**.**	**C**	**G**	**T**	**C**	**C**	**T**	**T**	**T**	**T**	**T**	**T**	**T**	**C**	**G**	**.**	**.**	**T**	**T**	**T**	**A**	**G**	**C**
TT Cytb5	T	G	C	A	C	T	C	A	.	C	G	T	C	C	T	T	T	T	T	T	T	C	G	.	.	T	T	T	A	G	C
TT Cytb12	T	G	C	A	C	T	C	A	.	C	G	T	C	C	.	.	T	T	T	T	T	C	.	C	C	T	T	T	A	G	C
TT Cytb14	T	G	C	A	C	T	C	A	.	C	G	T	C	C	T	T	T	T	T	T	T	C	G	.	.	T	T	T	A	G	C

																										1	1	1	1	1	1
	5	6	6	6	6	6	6	6	6	6	7	7	7	7	7	8	8	8	8	8	8	8	8	9	9	0	0	0	0	0	0
	9	1	3	4	5	6	7	8	8	9	2	2	3	5	9	1	3	4	5	6	6	7	7	6	7	1	3	3	4	7	7
	2	3	4	8	5	4	4	0	6	5	1	7	9	1	3	2	5	4	0	2	8	0	1	8	0	2	6	7	5	4	8
***Tursiops australis*** ** sp. nov.**																															
**Burru Cytb1 holotype**	**T**	**C**	**T**	**C**	**T**	**T**	**C**	**C**	**C**	**C**	**G**	**T**	**T**	**T**	**C**	**T**	**T**	**A**	**A**	**C**	**T**	**C**	**T**	**C**	**G**	**C**	**T**	**A**	**C**	**C**	**T**
Burru Cytb1	.	.	.	.	.	.	.	.	.	.	.	.	.	.	.	.	.	.	.	.	.	.	.	.	.	.	.	.	.	.	.
Burru Cytb3	.	.	.	.	.	.	.	.	.	.	.	.	.	.	.	.	.	.	.	.	.	T	.	.	.	.	.	.	.	.	.
Burru Cytb4	.	.	.	.	.	.	.	.	.	.	.	.	.	.	.	.	.	.	.	.	.	T	.	.	.	.	.	.	T	.	.
***Tursiops truncatus***																															
***T. maugeanus*** ** TTCytb5 lectotype**	**C**	**T**	**C**	**T**	**C**	**C**	**T**	**T**	**T**	**T**	**A**	**C**	**C**	**C**	**T**	**C**	**C**	**C**	**G**	**.**	**.**	**T**	**C**	**T**	**A**	**T**	**.**	**.**	**.**	**T**	**C**
TT Cytb5	C	T	C	T	C	C	T	T	T	T	A	C	C	C	T	C	C	C	G	.	.	T	C	T	A	T	.	.	.	T	C
TT Cytb12	C	T	C	T	C	C	T	T	T	T	A	C	C	C	T	.	C	C	G	G	C	T	C	T	A	T	.	C	.	.	.
TT Cytb14	C	T	C	T	C	C	T	T	T	T	A	C	C	C	T	C	C	C	G	.	.	T	C	T	A	T	C	.	.	T	C

Diagnostic sites separating south-east Australian *Tursiops australis* sp. nov. (Burru) and *T. truncatus* (TT) for mtDNA cytochrome *b*, 1086 base sequence. *Tursiops australis* sp. nov. holotype given as the reference sequence. Eighteen samples identified six unique haplotypes, three *Tursiops australis* sp. nov. and three *T. truncatus*, defined by 62 variable sites (59 transition substitutions, 3 transversion substitutions). Forty-eight fixed site differences were noted between the two species.

### Control region

A 418-base sequence of the mtDNA control region was obtained from 21 samples representing both cranial and external morphology groups (Table S9). Eight unique haplotypes were identified, three representing *T. australis* sp. nov. (two of which have previously been reported [13]) and five representing *T. truncatus*, defined by 30 variable sites (25 transition substitutions, five transversion substitutions and one single based insertion/deletion), when also including haplotypes previously reported in Charlton et al [13]. Ten diagnosable fixed base pair differences were found between the species (Table 5). In a similar way to the cytochrome *b* region, *T. australis* sp. nov. showed less intra-specific variation (three variable sites) when compared to *T. truncatus* (13 variable sites). Genetic sequences from the current study have been deposited on GenBank (Table S10).

**Table 5:** Mitochondrial DNA control region diagnostic sites.

										1	1	2	1	1	1	1	2	2	2	2	2	2	2	2	2	2	2	3	3	3
		1	2	6	6	7	7	7	9	0	1	5	6	7	7	9	0	1	2	4	4	4	4	4	5	5	6	3	7	7
	7	3	9	1	9	0	2	3	3	7	2	2	8	8	9	7	8	2	8	0	1	2	5	6	7	8	6	3	0	1
***Tursiops australis*** ** sp. nov.**	**T**	**T**	**T**	**C**	**C**	**G**	**A**	**T**	**C**	**C**	**T**	**T**	**C**	**C**	**A**	**T**	**C**	**A**	**A**	**T**	**T**	**T**	**T**	**A**	**C**	**C**	**A**	**T**	**A**	**A**
**holotype Burru CR6**																											**.**			
BurruCR2	.	.	.	.	.	.	.	.	.	.	.	.	T	.	.	.	.	.	.	.	.	.	.	.	.	.	.	.	.	.
BurruCR6	.	.	.	.	.	.	.	.	.	.	.	.	.	.	.	.	.	.	.	.	.	.	.	.	.	.	.	.	.	.
Burru CR8	.	.	.	.	.	.	.	.	T	.	.	.	.	.	.	.	.	.	.	A	.	.	.	.	.	.	.	.	.	.
Burru CR1*	.	.	.	.	.	.	.	.	.	.	.	.	.	.	.	.	.	.	.	.	.	.	.	.	.	T	.	.	.	.
BurruCR3*	.	.	.	.	.	.	.	.	.	.	.	.	.	.	.	.	.	.	.	.	.	.	.	.	.	.	G	.	.	.
BurruCR7*	.	.	.	.	.	.	.	.	T	.	.	.	.	.	.	.	.	.	.	.	.	.	.	.	.	.	.	.	.	.
***Tursiops truncatus***																														
***T. maugeanus*** ** lectotype CRTT29**	**C**	**.**	**.**	**T**	**.**	**A**	**G**	**C**	**.**	**T**	**.**	**C**	**.**	**T**	**G**	**.**	**T**	**C**	**C**	**.**	**C**	**C**	**C**	**C**	**.**	**.**	**.**	**.**	**-**	**G**
CRTT1	C	A	.	T	.	A	G	C	.	T	.	.	.	T	G	.	T	C	C	.	C	C	C	C	.	.	.	.	-	G
CRTT2	C	.	C	T	.	A	G	C	.	T	.	.	.	T	G	.	T	C	C	.	C	C	C	C	.	.	.	.	-	G
CRTT14	C	.	.	T	.	A	G	.	.	T	C	.	.	T	.	C	.	C	C	.	.	.	.	C	T	.	.	C	-	.
CRTT28	C	.	.	T	T	A	G	.	.	T	C	.	.	T	.	C	.	C	C	.	.	.	.	C	T	.	.	C	-	.

Diagnostic sites separating south-east Australian *Tursiops australis* sp. nov. (Burru) and *T. truncatus* (CRTT) for mtDNA control region, 418 base sequence. *Tursiops australis* sp. nov. holotype given as the reference sequence. Twenty-one samples identified eight unique haplotypes, three *Tursiops australis* sp. nov. and five *T. truncatus*, defined by 30 variable sites (25 transition substitutions, 5 transversion substitutions and one single based insertion/deletion); with the inclusion of four additional *T. australis* haplotypes (*) from Charlton et al. (2006). Ten diagnosable fixed site differences were found between the species.

### Concordance between morphological and molecular groups

In order to assess which species the morphological ‘groups’ genetically represented, the individual's mitochondrial DNA haplotype were overlaid on both cranial and external morphological datasets (samples highlighted by ★ in Figure 1a & 2a). The two distinct ‘groups’ from south-eastern Australian specimens concurred perfectly with *Tursiops australis* sp. nov. (Group 1) and southern form *T. truncatus* (Group 2) (Figure 1 & 2).

### Tursiops maugeanus specimens

**Figure 3:**
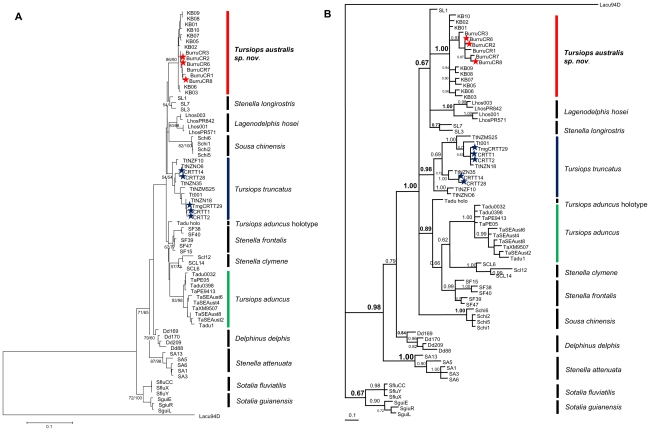
Phylogenetic analysis of the mtDNA control region haplotypes (A–B). Haplotypes specific to the study are denoted by ★ red = *Tursiops australis* sp. nov., blue = *T. truncatus*. (**A**) Consensus tree obtained by Maximum Likelihood and Neighbour-joining methods from mtDNA control region haplotypes. Tree is rooted with the outgroup *Lagenorhynchus acutus*. Bootstrap values >50% are indicated (1000 replicates: ML left value; NJ right value)(intra-species specific values not reported). (**B**) Majority rule consensus tree from Bayesian reconstruction (MrBayes) with posterior probabilities branch support values.

As stated, cranial and external morphology analyses presented several distinct groups. In all cases Group 1 incorporated the female *T. maugeanus* specimen (QVMAG#1365) and Group 2 incorporating the male *T. maugeanus* specimen (QVMAG#1360) (Figure 1a). MtDNA sequences (cytochrome *b* and control region) place the female *T. maugeanus* (QVMAG 1365) within *Tursiops australis* sp. nov. and the male *T. maugeanus* holotype (QVMAG 1360) within *T. truncatus* (Table 4 and 5; Figure 3).

### Phylogenetic analyses

Phylogenetic reconstructions by Maximum Parsimony (MP), Neighbour-joining (NJ) and Bayesian analyses showed *Tursiops australis* sp. nov. clearly distinct from both *Tursiops* species, and in a monophyletic clade outside of any reported genera (Figure 3a & b). MP and NJ analysis methods showed very similar topologies, with minor discrepancies overall. As such we present here a consensus tree of the ML and NJ analysis of the mtDNA control region (Figure 3a). The tree was characterised by low level of resolution for most nodes, however bootstrap support for differentiation of each species was more robust (Firgure3a). Bayesian inference analysis showed only one slight variation in topology with the placement of *Sousa chinensis* (Figure 3b). Both ML and NJ phylogenetic reconstruction show a sister relationship to *Stenella longirostris*, whilst Bayesian analyses showed a sisters relationship to *S. longirostris* and also *Lagenodelphis hosei*.

### Nomenclatural Acts

The electronic version of this document does not represent a published work according to the International Code of Zoological Nomenclature (ICZN), and hence the nomenclatural acts contained in the electronic version are not available under that Code from the electronic edition. Therefore, a separate edition of this document was produced by a method that assures numerous identical and durable copies, and those copies were simultaneously obtainable (from the publication date noted on the first page of this article) for the purpose of providing a public and permanent scientific record, in accordance with Article 8.1 of the Code. The separate print-only edition is available on request from PLoS by sending a request to PLoS ONE, Public Library of Science, 1160 Battery Street, Suite 100, San Francisco, CA 94111, USA along with a check for $10 (to cover printing and postage) payable to “Public Library of Science”.

In addition, this published work and the nomenclatural acts it contains have been registered in ZooBank, the proposed online registration system for the ICZN. The ZooBank LSIDs (Life Science Identifiers) can be resolved and the associated information viewed through any standard web browser by appending the LSID to the prefix “http://zoobank.org/”. The LSID for this publication is urn:lsid:zoobank.org:pub:9A469754-EFA0-499E-AF4C-772331B34025.

#### TAXONOMIC TREATMENT

In our quantitative and qualitative morphological and molecular comparisons of the designated southern form *T. maugeanus* syntypes together with specimens from numerous strandings over the past century, it is clear that the two syntype specimens of *T. maugeanus* comprise two different species (Figure 1a; Table 4 & 5). In all cases, morphological and molecular, the male (QVMAG 1360) concurred with the southern hemisphere *T. truncatus*, while the female (QVMAG 1365), concurred with the undescribed species, SABD.

To clarify the taxonomy of *T. maugeanus* we identified two alternative taxonomic treatments. One option was to designate the female as the lectotype of *T. maugeanus* and thus resurrect the name; with this option, the paralectotype male would simply be subsumed under *T. truncatus*. However, Scott's handwriting on the specimen label of the male, as well as his own extensive published and unpublished notes (held at QVMAG), make it clear that the species he envisaged was based on the male. Therefore, and given the current uncertain state of relationships within Delphininae [4], [5], [6], if it is someday demonstrated that the southern form of *T. truncatus* sensu Scott and Lord [19] and Iredale and Troughton [21] is distinct from the northern form, and if the female retained the name, our action would leave the name *T. maugeanus*, in essence, assigned to the wrong form. The other option was to designate the male as the lectotype of *T. maugeanus*, and leave it as a questionable junior synonym of *T. truncatus* for now; with this option, the paralectotype female would then be left without an identity.

After considerable consultation, we are convinced that the most conservative and stable approach is the second option above, thus leaving *T. maugeanus* as the appropriate available name for the southern form of *T. truncatus*, should it be found distinct from the northern. We anticipate that this is likely to occur, given the historical conclusions [19], [21] and in light of the recent designation of the *T. aduncus* holotype [34] whereby the South African Indo-Pacific form would be the name bearer of *T. aduncus*, leaving the western Pacific/Southeast Asian form *T. aduncus* possibly requiring a new name [34]. This then leaves the female paralectotype specimen, and the species she represents, needing a formal identity and thus also becomes available to be the holotype of the new species.

**REVISED TAXONOMY OF** ***TURSIOPS MAUGEANUS*** **as** ***TURSIOPS. TRUNCATUS***

Order Cetacea Brisson, 1762

Family Delphinidae Gray, 1821

Subfamily Delphininae *sensu* LeDuc, 1999

Genus *Tursiops* Gervais, 1855

***Tursiops maugeanus*** **Iredale and Troughton, 1934**

### Synonomy

*Tursiops tursio*, southern form male. – Scott and Lord 1919: 96, pl. XXIII–XXV [in part]

*Tursiops maugeanus* - Iredale and Troughton 1934: 68, nom. nov. for *T. tursio* (southern form) Scott and Lord 1919 [in part].

*Tursiops truncatus* - Möller et al. 2008: 676; Kemper 2004: 42; Perrin 2009.

### Lectotype here designated

QVMAG 1360, Cataract Gorge, Launceston, Tasmania, Australia, 1902.

### Paralectotype

QVMAG 1365, Hobblers Bridge, North Esk River 5 km upstream from Tamar River, 11 November 1914; misidentified and does not in fact belong to *T. maugeanus* ( = southern form of *T. tursio*), but represents an entirely new form requiring a separate name.

### Revised Diagnosis

**Figure 4:**
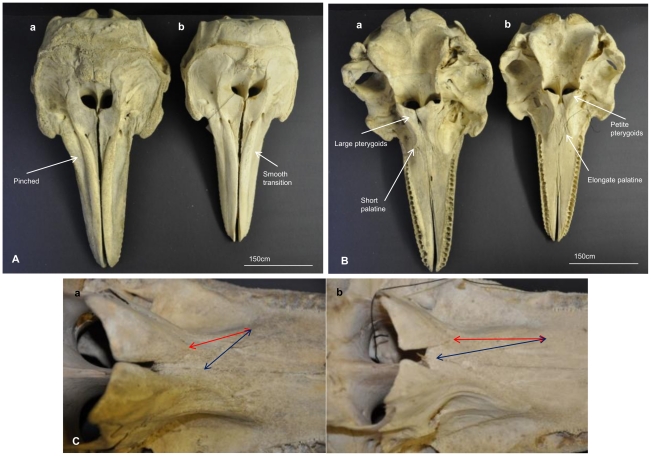
Direct visual comparison of cranial morphology (A–C). *Tursiops truncatus* (**a:** representing Group 2 of multivariate analyses QVMAG#1360, as lectotype of *T. maugeanus*); and *Tursiops australis* sp. nov. (**b:** representing Group 1of multivariate analyses QVMAG #1365, holotype). (**A**) Skulls are shown in dorsal view, note maxilla- premaxilla. (**B**) Ventral view, location of pterygoids and palatine noted (shown magnified in **C**). (**C**) Views of the pterygoids and palatine regions red = palatine length, blue = palatine suture angle.

Body large, robust (mean 2.95 m in length; range 2.83–3.02 m); with a short rostrum (mean 11.6 cm; range 11–12.5 cm); with tall and falcate dorsal fin (mean 29 cm in height; range 23.5–34 cm); with two-banded colouration dorsally slate grey-black, ventrally off-white; lacking a pale shoulder blaze and ventral spotting. Skull is large and robust (mean 527.88 mm; range 505.5–547 mm), the rostrum is short (mean 143.05 mm; range 136.29–158.89 mm) and wide across all measures (Table 2), with shape of the suture between the palatine and maxilla being shallow triangular or flattened (mean 42.18 mm; range 23.88–57.84 mm); ratio between the pterygoids and palatine is approximately 2∶1; with obvious ‘pinched’ appearance where the maxilla transitions into the premaxilla (Figure 4). On average has 90 teeth (22 lower left; 22 lower right; 23 upper left; 23 upper right).

### Molecular diagnostic characters

See below comparison with *Tursiops australis* sp. nov.

### Remarks

Concordant results from multiple independent data sets suggest that the syntype specimens of *Tursiops maugeanus* belong to two different species (Figure 1; Table 4 & 5). The male lectotype specimen of *T. maugeanus* is identical in its morphological and molecular features to the offshore southern form of *T. truncatus* (Figure 1; Table 4 & 5). Therefore, we provisionally regard *T. maugeanus* as a junior synonym of *T. truncatus* under the current taxonomic system. If the southern form of *T. truncatus* (hereafter referred as s.f. *T. truncatus*) is demonstrated to be a different species or subspecies from the northern form, the appropriate available name for the southern form would be *T. maugeanus*. The female paralectotype, which does not belong to *T. maugeanus*, is treated below.

**TAXONOMY OF NEW SPECIES.**

Order Cetacea Brisson, 1762

Family Delphinidae Gray, 1821

Subfamily Delphininae *sensu* LeDuc, 1999

Genus *Tursiops* Gervais, 1855

***Tursiops australis*** **sp. nov.**

urn:lsid:zoobank.org:act:54BA663A-BDE6-4E12-A9D2-84F6793EF4EA

Figure 4 & 5

**Figure 5:**
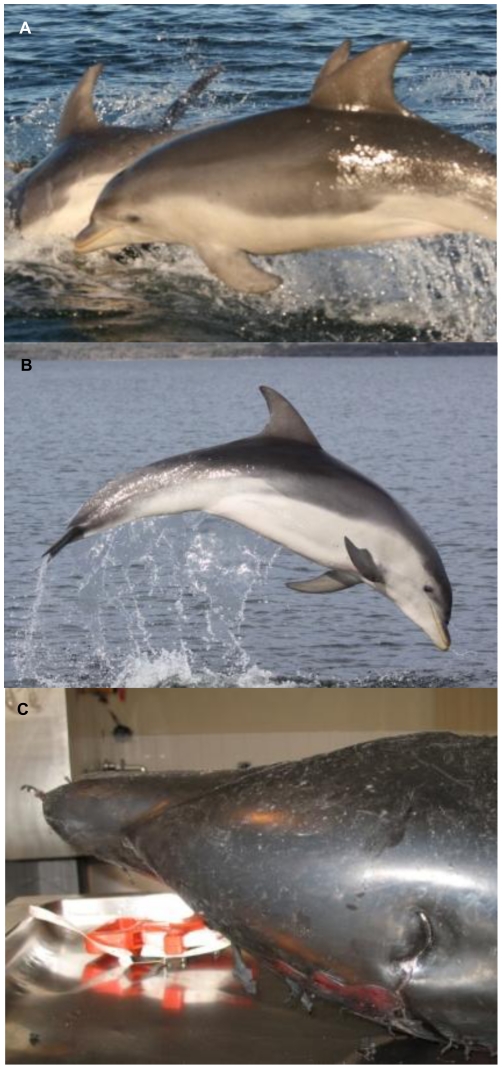
*Tursiops australis* sp. nov. external morphology and colouration (A–C). (**A and B**) Distinct tri-colouration, extension of ventrum white above eye, dorsal blaze, ‘stubby’ rostrum and falcate dorsal fin. (**C**) View of ‘stubby’ rostrum.

### Synonomy

*Tursiops tursio*, southern form; female. - Scott and Lord 1919: 96, pl. XXIII–XXV [in part].

*Tursiops maugeanus* - Iredale and Troughton 1937: 68, nom. nov. for *T. tursio* (southern form) Scott and Lord 1919 [in part].

*Tursiops* sp. - Scarpaci et al. 2003: 342; Warren-Smith and Dunn 2006: 357.

Victorian coastal bottlenose dolphin - Charlton et al. 2006: 173.

Southern Australian Bottlenose Dolphin - Möller et al. 2008: 676; Owen et al. 2011.

South Australian *T. truncatus* - Kingston et al. 2009:4.

*Tursiops truncatus* - Ross and Cockcroft 1990: 124.

*Tursiops aduncus* – Kemper 2004: 42.

Not *Tursiops truncatus* (Montagu, 1821): 75, pl. III.

Not *Tursiops tursio* (Fabricius, 1780): 49.

Not *Tursiops aduncus* (Ehrenberg, 1832)

### Etymology

Species name, *australis*, is in reference to the species link with Australia and is Latin for ‘southern’.

### Holotype

QVMAG 1365, Hobblers Bridge, North Esk River 5 km upstream from Tamar River, 11 November 1914; previously published as the female of the southern form of *Tursiops tursio* by Scott and Lord, 1919, later named *Tursiops maugeanu*s Iredale and Troughton, 1934. Repository location: Queen Victoria Museum and Art Gallery, Launceston, Tasmania, Australia.

### Paratypes

Monash MU210108, Beaumaris, Port Philip Bay, VIC, 21 Jan 2008; male. Monash MU230108, Point Henry, Geelong, VIC, 23 Jan 2008; male. MV C29579, Western Beach, Geelong, VIC, 14 Oct 1985; female. MV C29587, Kennedy's Point, Westernpoint Bay, VIC, 1 Jun 1992; male. MV C29667, Ocean Grove, VIC, 8 Jan 1987; female. MV C24944, Elwood, VIC, 2 Jun 1967; female. MV C28760, Sandringham, VIC, 13 Nov 1992; sex unknown. MV C29580, Murrells Beach, VIC, 17 Jan 1986; male. MV C29577, Safety Beach, VIC, 23 Jul 1985; female. MV C29586, Rippleside, VIC, 27 Jul 1991; male. MV C35986, Mitchell River, VIC, 4 Apr 2006; female. MV C35987, Hollands Landing, VIC, 21 Jul 2006. MV C35965, Lake Wellington, VIC, 14 Dec 2006; male. MV C35968, Poddy Bay, VIC, 30 Aug 2006; male. MV C35985, Blonde Bay, VIC, 1 Dec 2006; male. MV C35966, Lake Wellington, VIC, 14 Dec 2006; male. MV C36750, Paynesville, VIC, 4 Nov 2007; male. MV C29506, Sorrento, VIC, 16 Apr 1994; female. TMAG A1759, Marion Bay, TAS, 21 Feb 2003; unknown sex.

### Type Locality

North Esk River, 5 km upstream from Tamar River at Hobblers Bridge, Launceston, Tasmania (type locality), Port Phillip Bay and Gippsland Lakes, Victoria, Australia.

### Diagnosis

#### External morphology

*Tursiops australis* is smaller (mean 2.57 m in length; range 2.27–2.78 m) than s.f. *T. truncatus* (mean 2.95 m in length; range 2.83–3.02 m) (Table 3), larger than *T. adunc*us (mean 2.25 m in length; range 140–268 m [11]). Rostrum is smaller and ‘stubbier’ (mean 10.8 cm; range 9.4–12 cm) than *T. aduncus* (mean 13.4 cm; range 8.8–15.5 cm [11]), similar to s.f. *T. truncatus* (as above). Dorsal fin is falcate like *T. truncatus*, c.f. the small triangular fin of *T. aduncus*. *Tursiops australis* has a tri-banded colouration grading conspicuously as follows: dark bluish-gray dorsally and on the sides of the head and body; light gray along the midline, extending as a pale shoulder blaze on the flank below the dorsal fin; and off-white ventrally, extending over the eye and above the flipper in some individuals; without ventral spotting (Figure 5).

### Skull morphology

The skull is more ‘petite’ than s.f. *T. truncatus*. Average skull length (CBL) is 493.58 mm (range 470–513 mm) smaller than that of s.f. *T. truncatus* (as above) and larger than *T. aduncus* (mean 441 mm; range 424–455 mm). Across all measures the rostrum is wider and shorter than *T. aduncus* (Table 2). The palatine is long (mean 55.98 mm; range 45.27–63.85 mm) and the shape of the suture between the palatine and maxilla is an elongated triangular shape, in contrast to s.f. *T. truncatus* and *T. aduncus* shallow triangular or flattened shape (mean 42.18 mm; range 23.88–57.8 mm and mean 41.42; range 31.36–47.13 respectively) (Table 2 and Figure 4). The ratio between the pterygoids and palatine observed in *T. australis* is approximately 1∶1, c.f. 2∶1 for s.f. *T. truncatus*. On average *T. australis* has 94 teeth (23 lower left; 23 lower right; 24 upper left; 24 upper right). Teeth are long and conical, with older Gippsland Lakes and Tasmanian animals exhibiting substantial wear in the front and back teeth. The maxilla is flattened and smoothly transitional into the premaxilla toward the base of the rostrum, lacking the obvious the ‘pinched’ appearance of s.f. *T. truncatus* (Figure 4).

### Molecular diagnostic characters

*Tursiops australis* differs from s.f. *T. truncatus* significantly at 58 diagnosable fixed base pairs across two mtDNA gene regions, 48 fixed site differences in a 1086-base sequence of the mtDNA cytochrome *b* region (Table 4) and 10 differences along a 418-base sequence of the mtDNA control region (Table 5).

### Common Name

We propose the common name ‘Burrunan Dolphin’ for *Tursiops australis*. ‘Burrunan’ is an Australian aboriginal name given to dolphins (used in the Boonwurrung, Woiwurrung and Taungurung languages) meaning “*name of a large sea fish of the porpoise kind*” [47]. One of the two only known resident populations of *T. australis* is in Port Phillip Bay where the Boonwurrung people have documented their existence for over 1000 years.

### Distribution

South-eastern and southern Australian coastal waters, including Victoria, Tasmania and South Australia (Figure S1).. Two known resident populations of *T. australis* occur in Victoria; Port Phillip Bay (est. 90 animals [26]) and the Gippsland Lakes [13] where we estimate ∼50 animals). *Tursiops australis* haplotypes have also been documented from dolphins in eastern Tasmanian waters [14] and coastal regions of South Australia in the Spencer Gulf region and west to St. Francis Island [14], [48]. No *T. australis* haplotypes have been reported north of the Victoria/New South Wales border, or west of St. Francis Island, South Australia.

## Discussion

Here we present clear and consistent molecular and morphological differences thus demonstrating the existence of a new species of dolphin in south-eastern Australian waters.

### Relationships of Tursiops australis with other taxa

Morphological analyses reveal the new species and the two recognised *Tursiops* species differ in quantitative and qualitative cranial characters and in external morphology. The *combination* of overall size of the adult body, rostrum length and width, tall and falcate dorsal fin, the distinctive tri-colouration patterning and the extension of the white ventrum extending over the eye in *T. australis* (Figure 5; Table 3) differ conspicuously from the two recognised *Tursiops* species in Australian waters.

Cranial comparisons between *T. australis* and *T. truncatus* from south-eastern Australia and *T. aduncus* from Queensland, Australia (current study) show significant differences across multiple measures (Table 2). The three species grouped separately using multiple forms of statistical analyses (Figure 1 and S2). *Tursiops australis* overall size and shape of the skull is somewhat intermediary between the two recognised *Tursiops* species, however there are only a few characters that overlap in their range (Table 2). Two particular qualitative cranial characters, the shape of the suture between the palatine and maxilla (quantifiable by a ratio between the length of the pterygoids and palatine), and the smooth transition between the maxilla and pre-maxilla region (Figure 4) are clearly diagnostic of *T. australis*. When comparing *T. australis* to *T. aduncus* there is also clear differences. *Tursiops australis* shows a longer and wider skull to *T. aduncus* holotype specimen [34] and to reported *T. aduncus* from both South African and Chinese water [32] (Table S11). In addition the *T. aduncus* rostrum is significantly narrower across all measures and has more teeth (Table 2 and Table S11).

Further, animals grouped by external and cranial morphometrics as either *T. australis* or s.f. *T. truncatus* were in every case identified to the same group determined using molecular analysis (Figure 1). Charlton et al. [13] found high mtDNA control region sequence divergence between the new species and *Tursiops truncatus* (5.5%) and between the new species and *T. aduncus* (9.1%). Using mtDNA cytochrome *b*, Möller et al. [14] reported between 5.5% and 7.7% divergence between the new species and *T. truncatus*. This is larger than between *T. truncatus* and *T. aduncus* (3.2%–5.8%) [14], and between several other delphinid species that are grouped in the same genus, such as between *Lagenorhynchus obscurus* and *L. obliqidens* (1.22%) [49], *Delphinus delphis* and *D. capensis* (1.09%) [50], and between the recently described *Sotalia fluviatilis* and *S. guianensis* (2.5%) [2], and *Orcaella heinsohni* and *O. brevirostris* (5.9%) [3]. We show this divergence is supported by clear diagnostic fixed sequence differences between *T. australis* and s.f. *T. truncatus* (cytochrome *b* = 48 fixed differences; control region = 10 fixed differences; Table 4 and 5 respectively). In addition, Möller et al [14] examined the new species (designated mtDNA clusters) using multiple nuclear markers and found evidence for complete reproductive isolation of the new species to both *T. truncatus* and *T. aduncus*. This high level of genetic divergence, complete reproductive isolation and the ambiguity of placement within any recognised genera strongly indicate that these coastal dolphins are not simply ecotypes of either recognised *Tursiops* species but are in fact representative of a new species. Irreversible divergence and distinct evolutionary trajectory of *T. australis* from recognised *Tursiops* species appears indisputable based on these multiple non-overlapping data sets.

### Polyphyly of Tursiops

As previously discussed, numerous studies have demonstrated that *Tursiops* is polyphyletic [5], [12], [13], [14], [15]. When assessing the phylogenetic relationships of *T. aduncus*, using the mtDNA cytochrome *b* and the control region, the sister taxa most commonly suggested is *D. delphis* and *S. coerueloalba* [5], [13], [14], in addition Möller et al. [14] has also suggested *S. clymene* and *S. frontalis* are also sister taxa. However based on mtDNA control region Kingston et al. [51] does not show the sister relationship between *D. delphis* and *T. aduncus* and based on AFLP places *T. aduncus* with *L. hosei* (using Nei-Lei neighbour joining anlaysis). Regardless of the DNA region used and phylogenetic analysis performed *T. truncatus* forms a separate clade from *T. aduncus* [5], [12], [13], [14], [51].

In this study we have also shown that *Tursiops* in polyphyletic, with *T. aduncus*, *T. truncatus* and *T. australis* on three independent lineages. Using all three phylogenetic analyses, the placement of *T. australis* is outside of both *Tursiops* species, with a sister relationship to *S. longirostris* (using ML and NJ methods) and additionally *L. hosei* using Bayesian inferences. Möller et al. [14] using mtDNA cytochrome *b* suggests the same sister relationships.

### Alternative taxonomies

Unlike other recently recognised species, *Orcaella heinsohni* [3] and *Sotalia guianensis* [2] where the classification within pre-existing genera was clear, this species based on multiple molecular regions [4], [13], [14] does not associate unambiguously with any existing genus. Whilst, as the discussed, the genus *Tursiops* is currently accepted as polyphyletic, Kingston et al. [4] states there is no support for a close genetic relationship between the two recognised *Tursiops* species, despite the morphological similarities, and along with others, calls on a review of not only the genus *Tursiops* but of family Delphinidae [4], [5], [12]. Natoli et al. [12] also raising the issue of generic affinities of *Tursiops*, more specifically the South African *adunucus*-type with the reported closeness to *D. delphis*, however no attempt was made at resolving the generic affinities. Given this current state of taxonomic uncertainty we believe that the most conservative approach at this time is to classify the new species in genus *Tursiops*, pending revision. We further believe that once revision of the Delphinidae is conducted, it is likely that this new species will be shown to represent a unique genus; if that is the case, we believe the genus name *Tursiodelphis* would be appropriate (from the Latin ‘tursio’, meaning ‘porpoise’, and Greek ‘delphis’, meaning ‘dolphin’).

In contrast, a number of nuclear DNA regions were also investigated in this study (data not shown). They include intron regions; CHRNA (283 bp) and POLA (330 bp) [52] and anonymous nuclear regions; Del10 (346 bp), Del 12 (575 bp) and Del 16 (533 bp) [53] for 19 individuals from *T. australis*, *T. truncatus*, *T. aduncus* and *L. hosei* (with species identification based on mtDNA regions). Of the five intron regions, four suggested no differentiation between the four species however one region (Del12) showed consistent species specific differences, defined by 3 variable sites (all transition substitutions). A possible explanation for this lack of differentiation may be due to the slower evolving nuclear regions, the rapid radiation of the delphinids (as also highlighted also by the current confused state of many generic affinities of dolphins [5], [12], [15]) and thus the potential of recent shared ancestry of these species. Caballero et al. [15] found significantly less parsimonious informative characters at each of the nine intron regions in comparison to each of the mtDNA control region and cytochrome *b*. In addition, the small samples size, taxa examined and lack of available Delphinidae GenBank submitted intron sequences for comparison may also be the limiting factor for species differentiation in this case. Larger sample sizes and greater representation from multiple taxa across Delphinidae would clearly be required to investigate generic affinities further, however, the clear and consistent morphological and molecular differentiation presented in this paper clearly support species level distinction.

### Additional evidence

An additional line of evidence for separation of *T. australis* and s.f. *T. truncatus* is provided in Owen et al. [27] using stable isotope signatures of both species. Owen et al. [27] indicated *T. australis* (noted as SABD) was distinct from s.f. *T. truncatus* (noted as common bottlenose dolphin CBD), with s.f. *T. truncatus* having significantly lower values for δ^13^C and δ^15^N compared to *T. australis*. They conclude this distinction of the stable isotope signatures between the two species strongly indicates they forage in different areas and are likely to feed on different prey, thus providing an additional line of evidence for the recognition of *T. australis*.

### Conservation value

Whilst, as previously discussed, there is an urgent requirement to undertake a full review of the Delphinidae Family, this manuscript is an important step in this review. This new species has erroneously been ‘labelled’ *Tursiops truncatus* and *T. aduncus*, of which we have demonstrated with clear and consistent evidence that it is neither. In addition, these dolphins have been ‘living under the eye’ in a well populated urban environment, have been the focus of multiple researchers and due to the multi-disciplinary approach taken in the manuscript we have been able to formally identify this species, thus highlighting the ‘need’ for other such studies to not ‘look in isolation’ of one line of evidence but to use a multiple disciplinary approach to assess the level of divergence.

The formal recognition of this new species is of great importance to correctly manage and protect this species, and has significant bearing on the prioritization of conservation efforts. This is especially crucial given it's endemism to a small region of the world, with only two small known resident populations and the proximity of those to major shipping ports, commercial and recreation fisheries, residential, industrial and agricultural stressors. Recognition of this new species opens the pathway that *T. australis* would qualify for listing as a threatened species under the Australian *Environment Protection and Biodiversity Conservation Act 1999* (EPBC Act) thus allowing immediate and directed conservation effort for further protection.

## Supporting Information

Figure S1 Sampling locations of dolphins across Australia
(TIF)

Figure S2 Principal component analysis on cranial measures; scatter plot of Principal components 1 & 2
(TIF)

Figure S3 Principal component analysis on external morphology measures; scatter plot of Principal components 1 & 2
(TIF)

Table S1 Cranial measures
(DOC)

Table S2 External morphology measures
(DOC)

Table S3 Principal component analysis loadings of the first three Principal components (PC) on 34 cranial measures from 40 ‘bottlenose’ dolphin skulls
(DOC)

Table S4 Principal component analysis loadings of the first three Principal components (PC) for 11 external measures from 17 ‘bottlenose’ dolphins
(DOC)

Table S5 PCR primers used to amplify mtDNA cytochrome *b* gene and control region from *Tursiops maugeanus* syntype specimens
(DOC)

Table S6 GenBank accession numbers and species information for samples incorporated to phylogenetic analyses
(DOC)

Table S7 Discriminant function analysis loadings on 34 cranial measures from 40 ‘bottlenose’ dolphin skulls
(DOC)

Table S8 Discriminant function analysis loadings for 11 external measures from 17 ‘bottlenose’ dolphins
(DOC)

Table S9 Species classification overview based on different analyses and characters
(DOC)

Table S10 GenBank accession numbers of the sequences from this study
(DOC)

Table S11 Average cranial measures (mm) and tooth counts for *Tursiops australis*, *Tursiops truncatus*, *Tursiops aduncus* (current study) and from *Tursiops aduncus* holotype (Perrin et al. 2007) and *Tursiops adunucs* (Wang et al. 2000)
(DOC)

## References

[1] Steeman ME, Hebsgaard MB, Fordyce RE, Ho SYW, Rabosky DL (2009). Radiation of Extant Cetaceans Driven by Restructuring of the Oceans.. Systematic Biology.

[2] Caballero S, Trujillo F, Vianna JA, Barrios-Garrido H, Montiel MG (2007). Taxonomic status of the genus Sotalia: Species level ranking for “tucuxi” (*Sotalia fluviatilis*) and “costero” (*Sotalia guianensis*) dolphins.. Marine Mammal Science.

[3] Beasley I, Robertson KM, Arnold P (2005). Description of a new dolphin, the Australian Snubfin dolphin *Orcaella heinsohni* sp. n. (Cetacea, Delphinidae).. Marine Mammal Science.

[4] Kingston SE, Adams LD, Rosel PE (2009). Testing mitochondrial sequence and anonymous nuclear markers for phylogeny reconstruction in a rapidly radiating group: molecular systematics of the Delphininae (Ceatcea: Odontoceti: Delphinidae).. BMC Evolutionary Biology.

[5] LeDuc RG, Perrin WF, Dizon AE (1999). Phylogenetic relationships among the delphinid cetaceans based on full cytochrome b sequences.. Marine Mammal Science.

[6] May-Collado L, Agnarsson I (2006). Cytochrome b and Bayesian inference of whale phylogeny.. Molecular Phylogenetics and Evolution.

[7] Tezanos-Pinto G, Baker CS, Russell K, Martien K, Baird RW (2009). A Worldwide Perspective on the Population Structure and Genetic Diversity of Bottlenose Dolphins (*Tursiops truncatus*) in New Zealand.. Journal of Heredity.

[8] Appeltans W, Bouchet P, Boxshall GA, Fauchald K, Gordon DP (2011). World Register of Marine Species.. http://www.marinespecies.org.

[9] Wang JY, Chou L-S, White BN (1999). Mitochondrial DNA analysis of sympatric morphotypes of bottlenose dolphins (genus: *Tursiops*) in Chinese waters.. Molecular Ecology.

[10] Wang JY, Chou LS, White BN (2000). Osteological differences between two sympatric forms of bottlenose dolphins (genus *Tursiops*) in Chinese waters.. Journal of Zoology.

[11] Wang JY, Chou L-S, White BN (2000). Differences in the external morphology of two sympatric species of bottlenose dolphins (genus *Tursiops*) in the waters of China.. Journal of Mammalogy.

[12] Natoli A, Peddemors VM, Hoelzel AR (2004). Population structure and speciation in the genus *Tursiops* based on microsatellite and mitochondrial DNA analyses.. Journal of Evolutionary Biology.

[13] Charlton K, Taylor AC, McKechnie SW (2006). A note on divergent mtDNA lineages of bottlenose dolphins from coastal waters of southern Australia.. Journal of Cetacean Research & Management.

[14] Möller LM, Bilgmann K, Charlton-Robb K, Beheregaray L (2008). Multi-gene evidence for a new bottlenose dolphin species in southern Australia.. Molecular Phylogenetics and Evolution.

[15] Caballero S, Jackson J, Mignucci-Giannoni AA, Barrios-Garrido H, Beltran-Pedreros S (2008). Molecular systematics of South American dolphins Sotalia: Sister taxa determination and phylogenetic relationships, with insights into a multi-locus phylogeny of the Delphinidae.. Molecular Phylogenetics and Evolution.

[16] Ross GJB, Cockcroft VG, Leatherwood S, Randall RR (1990). Comments on Australian Bottlenose Dolphins and the Taxonomic Status of *Tursiops aduncus* (Ehrenberg, 1832).. The Bottlenose Dolphin.

[17] Möller LM, Beheregaray LB (2001). Coastal bottlenose dolphins from southeastern Australia are *Tursiops aduncus* according to sequences of the mitochondrial DNA control region.. Marine Mammal Science.

[18] Krützen M, Sherwin WB, Berggren P, Gales N (2004). Population structure in an inshore cetacean revealed by microsatellite and mtDNA analysis: Bottlenose dolphins (*Tursiops* sp.) in Shark Bay, Western Australia.. Marine Mammal Science.

[19] Scott HH, Lord CE (1919). Studies of Tasmanian Cetacea. Pt 1.. Papers and Proceedings of the Royal Society of Tasmania.

[20] Kemper CM (2004). Osteological variation and taxonomic affinities of bottlenose dolphins, *Tursiops* spp., from South Australia.. Australian Journal of Zoology.

[21] Iredale T, Troughton E (1934). A check-list of the mammals recorded from Australia.. Memoirs of the Australian Museum.

[22] Perrin W (2009). World Cetacea Database.. http://www.marinespecies.org/cetacea.

[23] Australian Faunal Directory (2008). Species *Tursiops truncatus*.. http://www.environment.gov.au/biodiversity/abrs/online-resources/fauna/afd/taxa/Tursiops_truncatus.

[24] Hale PT (2002). Interactions between vessels and dolphins in Port Phillip Bay.

[25] Scarpaci C, Bigger SW, Saville TA, Nugegoda D (2000). The Bottlenose Dolphin Tursiops truncatus in the southern end of Port Phillip Bay: Behavioural characteristics in Spring and Summer.. Victorian Naturalist.

[26] Warren-Smith ÁB, Dunn WL (2006). Epimeletic Behaviour Toward a Seriously Injured Juvenile Bottlenose Dolphin (*Tursiops* sp.) in Port Phillip, Victoria, Australia.. Aquatic Mammals.

[27] Owen K, Charlton-Robb K, Thompson R (2011). Resolving the trophic relations of cryptic species: An example using stable isotope analysis of dolphin teeth.. PLoS ONE.

[28] Pichler FB, Robineau D, Goodall RNP, Meyer MA, Olivarria C (2001). Origin and radiation of Southern Hemisphere coastal dolphins (genus Cephalorhynchus).. Molecular Ecology.

[29] Reeves RR, Perrin WF, Taylor BL, Baker CS, Mesnick SL (2004). Report of the workshop on shortcomings of cetacean taxonomy in relation to needs of conservation and management.

[30] De Queiroz K (2007). Species Concepts and Species Delimitation.. Systematic Biology.

[31] Cipriano F, Rosel PE, Taylor BL, Hancock B, Robertson KM (2009). Using Genetic Evidence for recognizing Marine Mammal Subspecies.

[32] Wang JY, Chou L-S, White BN (2000). Osteological differences between two sympatric forms of bottlenose dolphins (genus *Tursiops*) in Chinese waters.. Journal of Zoology (London).

[33] Hale PT, Barreto AS, Ross GJB (2000). Comparative morphology and distribution of the aduncus and truncatus forms of bottlenose dolphin Tursiops in the Indian and Western Pacific Oceans.. Aquatic Mammals.

[34] Perrin WF, Robertson KM, Van Bree PJH, Mead JG (2007). Cranial description and genetic identity of the holotype specimen of Tursiops aduncus (Ehrenberg, 1832).. Marine Mammal Science.

[35] Inc. SS (2009). SYSTAT 13 for Windows Bangalore, India.

[36] Hammer O, Harper DAT, Ryan PD (2001). PAST: Paleontological statistic software package for education and data analysis.. 1.94b ed: Paleontologia Electronica.

[37] Suetin G, White BN, Boag PT (1991). Preservation of avian blood and tissue samples for DNA analysis.. Canadian Journal of Zoology.

[38] Krützen M, Barre LM, Möller LM, Heithaus MR, Simms C (2002). A biopsy system for small cetaceans: Darting success and wound healing in *Tursiops* spp.. Marine Mammal Science.

[39] Weber LI, Luca MJ, Barreto AS, Souza TT (2007). Successful Amplification of Mitochondrial DNA from Dentin of the Bottlenose Dolphin *Tursiops truncatus*.. Brazilian Archives of Biology and Technology.

[40] Shibata Y, Fujita S, Takahashi H, Yamaguchi A, Koji T (2000). Assessment of decalcifying protocols for detection of specific RNA by non-radioactive in situ hybridization in calcified tissues.. Histochem Cell Biol.

[41] Cooper A, Poinar H (2000). Ancient DNA: Do It Right or Not at All.. Science.

[42] Boessenkool S, Austin JJ, Worthy TH, Scofield P, Cooper A (2008). Relict or colonizer? Extinction and range expansion of penguins in southern New Zealand.. Proceedings Royal Society Biology.

[43] Tamura K, Peterson D, Peterson N, Stecher G, Nei M (2011). MEGA5: Molecular Evolutionary Genetics Analysis using Maximum Likelihood, Evolutionary Distance, and Maximum Parsimony Methods.. Molecular Biology and Evolution.

[44] Hasegawa M, Kishino H, Yano T (1985). Dating the human-ape split by a molecular clock of mitochondrial DNA.. Journal of Molecular Evolution.

[45] Swofford DL (2003). PAUP*. Phylogenetic analysis using parsimony (*and other methods)..

[46] Ronquist F, Huelsenbeck JP (2003). MrBayes3: Bayesian phylogenetic inference under mixed models.. Bioinformatics.

[47] Thomas W (1834). William Thomas Papers, Manuscript 214.. William Thomas Papers, Manuscript.

[48] Bilgmann K, Möller LM, Harcourt RG, Gibbs SE, Beheregaray LB (2007). Genetic differentiation in bottlenose dolphins from South Australia: association with local oceanography and coastal geography.. Marine Ecology-Progress Series.

[49] Hare MP, Cipriano F, Collins AG, Palumbi SR (2002). Genetic evidence on the demography of speciation in allopatric dolphin species.. Evolution.

[50] Rosel PE, Dizon AE, Heyning JE (1994). Gentic analysis of sympatric morphotypes of common dolphins (genus *Delphinus*).. Marine Biology.

[51] Kingston SE, Rosel PE (2004). Genetic differentiation among recently diverged delphinid taxa determined using AFLP markers.. Journal of Heredity.

[52] Lyons LA, Laughlin TF, Copeland NG, Jenkins NA, Womack JE (1997). Comparative anchor tagged sequences (CATS) for integrative mapping of mammalian genomes.. Nature Genetics.

[53] Amaral AR, Silva MC, Moller LM, Beheregaray LB, Coelho MM (2010). Anonymous nuclear markers for cetacean species.. Conservation Genetics.

