# Supplementary figures and images for: A New Dolphin Species, the Burrunan Dolphin Tursiops australis sp. nov., Endemic to Southern Australian Coastal Waters
Source: PLoS One. 2011 Sep 14;6(9):e24047. doi: 10.1371/journal.pone.0024047 (PMC3173360; doi:10.1371/journal.pone.0024047)

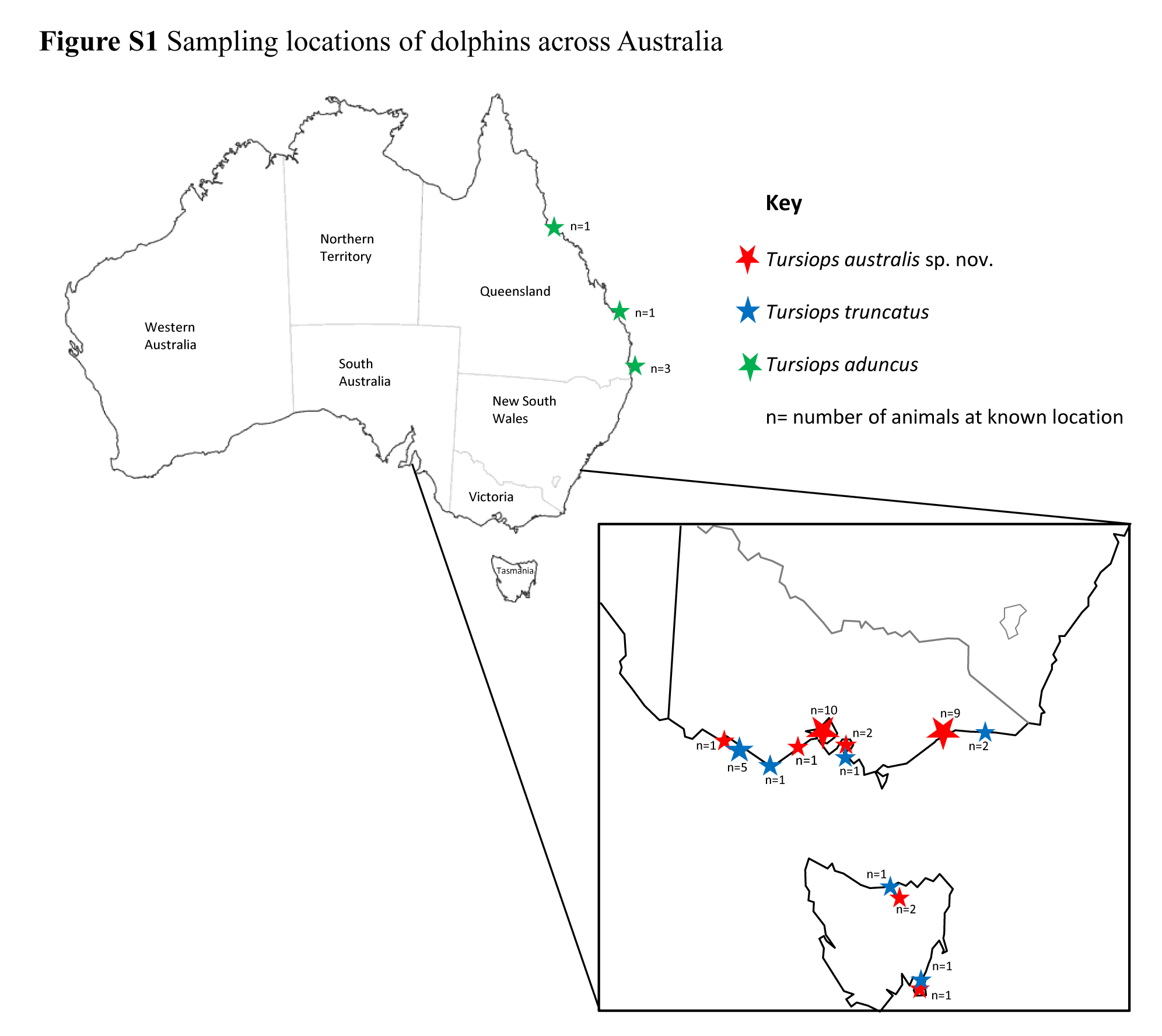

Supplement: Figure S1 — Sampling locations of dolphins across Australia (TIF) [file pone.0024047.s001.tif]

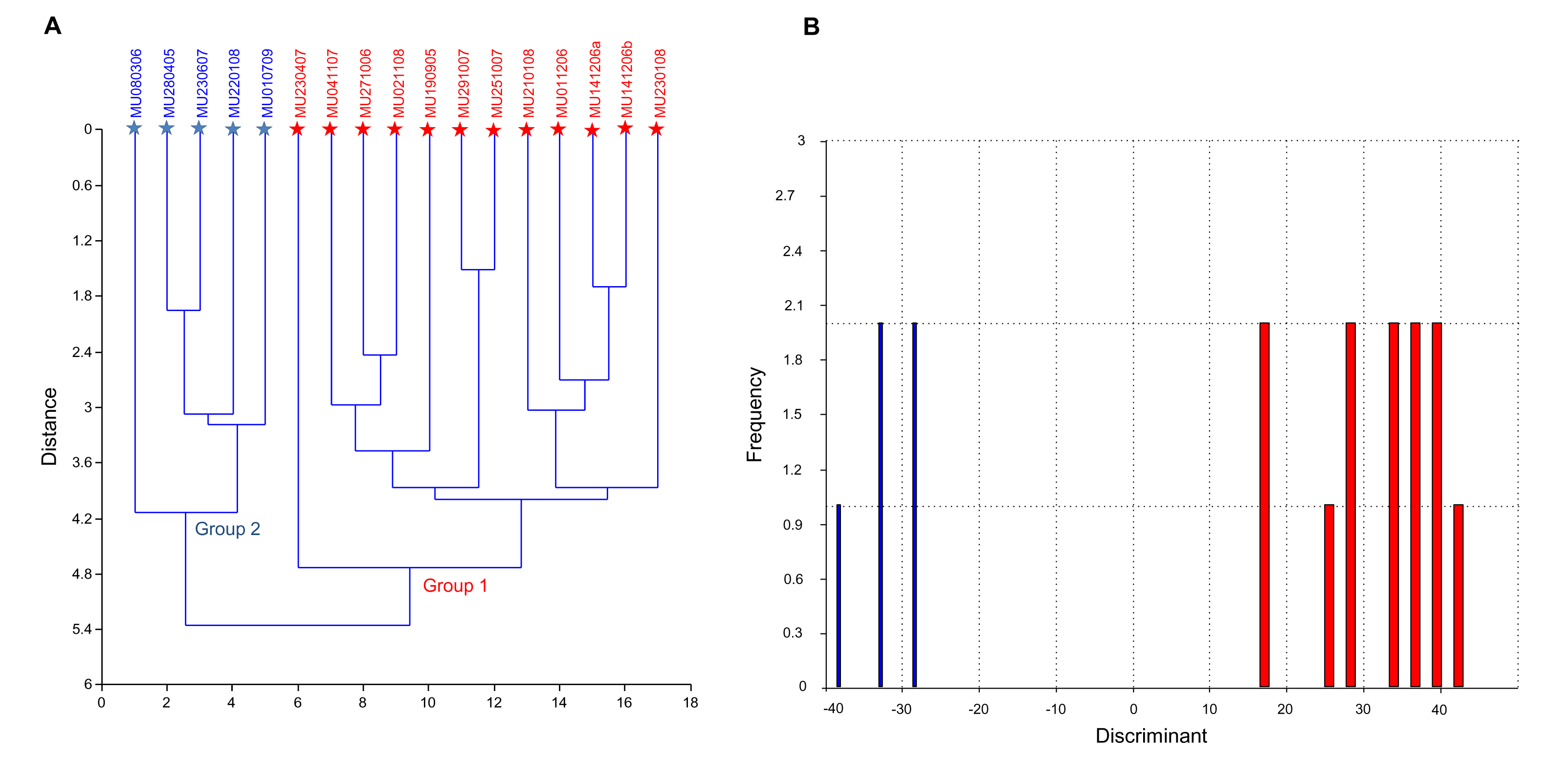

Supplement: Figure S2 — Principal component analysis on cranial measures; scatter plot of Principal components 1 & 2 (TIF) [file pone.0024047.s002.tif]

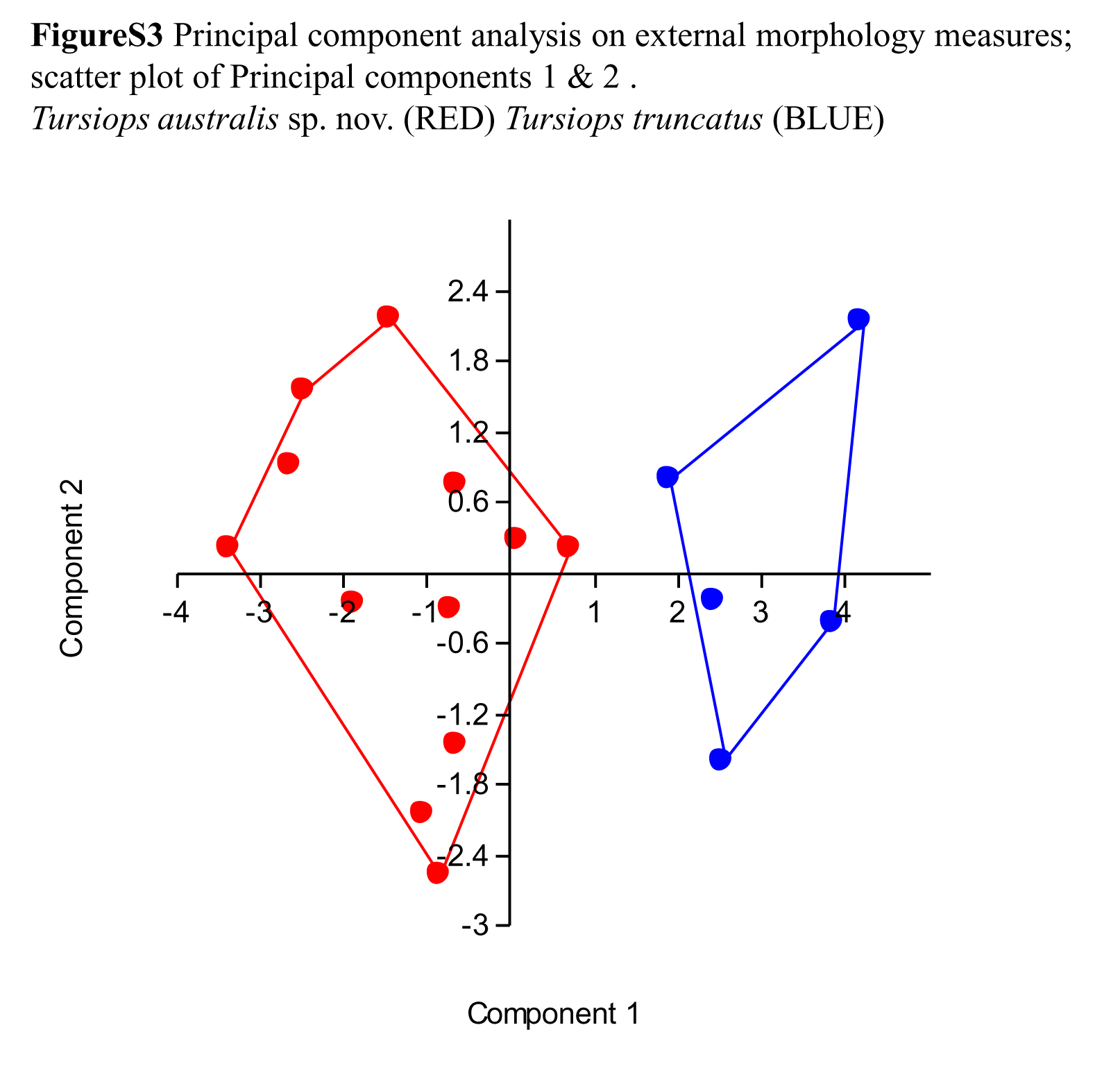

Supplement: Figure S3 — Principal component analysis on external morphology measures; scatter plot of Principal components 1 & 2 (TIF) [file pone.0024047.s003.tif]
