# Supplementary material for: A New Dolphin Species, the Burrunan Dolphin Tursiops australis sp. nov., Endemic to Southern Australian Coastal Waters
Source: PLoS One. 2011 Sep 14;6(9):e24047. doi: 10.1371/journal.pone.0024047 (PMC3173360; doi:10.1371/journal.pone.0024047)
Supplement: Table S1 — Cranial measures (DOC) [file pone.0024047.s004.doc]

**Table S1** Cranial measures

| **Measure code** | **Measure description** |  |
| --- | --- | --- |
| BL | bulla length |  |
| POL | periodic length |  |
| CBL | condylobasal length | |
| DFWN | dorsal frontal width at nasals | |
| DWFM | dorsal frontal width at maxilla | |
| GLPT | greatest length of the left pterygoid | |
| GLPTF | greatest length of the left temporal fossa | |
| GPARW | greatest width across parietals | |
| GPOW | greatest postorbital width | |
| GPRW | greatest preorbital width | |
| GWPTF | greatest width of left temporal fossa | |
| GWEN | greatest width of the external nares | |
| GWIN | greatest width of the internal nares | |
| GWPX | greatest width of the premaxillae | |
| LAL | length of the antorbital process of lacrimal | |
| LO | length of orbit |  |
| LTRL | lower tooth row length to tip of rostrum | |
| LWPTF | least width between posterior borders of temporal fossa | |
| MFL | mandibular fossa length | |
| MH | mandible height |  |
| ML | mandible length |  |
| MSL | mandibular symphysis | |
| PRW | premaxillae width at mid-rostral length | |
| RL | rostrum length |  |
| RWB | rostrum width at base | |
| RWM | rostrum width at mid-length | |
| RW60 | rostrum width at 60mm from base | |
| RW75 | rostrum width at three-quarters distance from base | |
| TREN | tip of the rostrum to external nares | |
| TRIN | tip of the rostrum to internal nares | |
| UTLTR | upper tooth row length to the tip of the rostrum | |
| ZW | zygomatic width of skull | |
| TPC | the tip of the rostrum to the apex of the premaxillary convexity | |
| VW | vomer width |  |
| WAS | width of alisphenoid at the suture with the basisphenoid | |
| APAP | anterior apex of pterygoids to palatine | |
| **Tooth counts** |  |  |
| TTLL | number of teeth on the lower left | |
| TTLR | number of teeth on the lower right | |
| TTUL | number of teeth on the upper left | |
| TTUR | number of teeth on the upper right | |
