# Supplementary material for: A New Dolphin Species, the Burrunan Dolphin Tursiops australis sp. nov., Endemic to Southern Australian Coastal Waters
Source: PLoS One. 2011 Sep 14;6(9):e24047. doi: 10.1371/journal.pone.0024047 (PMC3173360; doi:10.1371/journal.pone.0024047)
Supplement: Table S2 — External morphology measures (DOC) [file pone.0024047.s005.doc]

**Table S2 External morphology measures**

| **Measure code** | **Measure description** |
| --- | --- |
| UJAM | length, tip of upper jaw to apex of melon |
| UJGAP | length, tip of upper jaw to angle of gape |
| UJEYE | length, tip of upper jaw to centre of eye |
| UJBH | length, tip of upper jaw to blow hole |
| UJDF | length, tip of upper jaw to anterior insertion of dorsal fin |
| UJTDF | length, tip of upper jaw to tip of dorsal fin |
| TLEN | total length, tip of upper jaw to caudal notch |
| UJFLIP | length, tip of upper jaw to anterior insertion of flipper |
| UJGEN | length, tip of upper jaw to genital opening |
| UJANU | length, tip of upper jaw to centre of anus |
| LFLIP | length of flipper |
| WFLIP | width of flipper at maximum |
| WFLU | width of flukes |
| DCN | depth of caudal notch |
| HD | height of dorsal fin |
| PROJ | projection of lower jaw beyond upper |
| GIRMAX | girth at maximum |
| GIRANU | girth at anus |
