# Supplementary material for: A New Dolphin Species, the Burrunan Dolphin Tursiops australis sp. nov., Endemic to Southern Australian Coastal Waters
Source: PLoS One. 2011 Sep 14;6(9):e24047. doi: 10.1371/journal.pone.0024047 (PMC3173360; doi:10.1371/journal.pone.0024047)
Supplement: Table S3 — Principal component analysis loadings of the first three Principal components (PC) on 34 cranial measures from 40 ‘bottlenose’ dolphin skulls (DOC) [file pone.0024047.s006.doc]

**Table S3** Principal component analysis loadings of the first three Principal components (PC) on 33 cranial measures from 40 ‘bottlenose’ dolphin skulls

| **Cranial Character** | **PC1** | **PC2** | **PC3** |
| --- | --- | --- | --- |
| CBL | 0.978 | -0.008 | 0.016 |
| DFWN | -0.184 | 0.599 | 0.161 |
| DFWM | 0.148 | -0.518 | 0.423 |
| GLPTF | 0.561 | -0.211 | -0.416 |
| GWPTF | 0.586 | -0.323 | 0.055 |
| GWEN | 0.574 | -0.654 | 0.072 |
| GWIN | 0.900 | 0.119 | 0.001 |
| GPRW | 0.935 | 0.166 | -0.143 |
| GPOW | 0.949 | 0.165 | -0.120 |
| GWPX | 0.789 | -0.358 | -0.210 |
| LAL | 0.850 | 0.168 | -0.012 |
| LO | 0.456 | -0.439 | 0.378 |
| LTRL | 0.816 | 0.230 | 0.183 |
| LWPTF | 0.068 | -0.525 | 0.437 |
| MFL | 0.802 | -0.228 | -0.072 |
| MH | 0.889 | -0.070 | -0.040 |
| ML | 0.967 | 0.033 | 0.102 |
| MSL | 0.399 | 0.279 | 0.644 |
| PRW | 0.788 | -0.391 | -0.089 |
| RL | 0.934 | 0.169 | 0.065 |
| RWB | 0.923 | -0.223 | -0.080 |
| RW60 | 0.909 | 0.004 | -0.064 |
| RWM | 0.908 | -0.098 | -0.183 |
| RW75 | 0.827 | -0.068 | -0.184 |
| TREN | 0.936 | 0.132 | 0.075 |
| UTLTR | 0.909 | 0.043 | 0.165 |
| VW | 0.821 | -0.140 | 0.012 |
| ZW | 0.939 | 0.233 | -0.091 |
| APAP | -0.078 | -0.691 | -0.143 |
| TPC | 0.599 | 0.516 | -0.063 |
| WAS | 0.924 | 0.184 | 0.027 |
| TRIN | 0.943 | 0.068 | 0.200 |
| GLPT | 0.643 | 0.595 | 0.080 |
| Eigenvalues | 19.791 | 3.559 | 1.467 |
| % Total variance | 59.974 | 10.786 | 4.446 |
| **Cumulative % variance** | **59.974** | **70.760** | **75.206** |
