# Supplementary material for: A New Dolphin Species, the Burrunan Dolphin Tursiops australis sp. nov., Endemic to Southern Australian Coastal Waters
Source: PLoS One. 2011 Sep 14;6(9):e24047. doi: 10.1371/journal.pone.0024047 (PMC3173360; doi:10.1371/journal.pone.0024047)
Supplement: Table S4 — Principal component analysis loadings of the first three Principal components (PC) for 11 external measures from 17 ‘bottlenose’ dolphins (DOC) [file pone.0024047.s007.doc]

**Table S4** Principal component analysis loadings of the first three Principal components (PC) for 11 external measures from 17 'bottlenose' dolphins

| **External Measure** | **PC1** | **PC2** | **PC3** |
| --- | --- | --- | --- |
| UJGAP | 0.8968 | -0.01428 | 0.2771 |
| UJEYE | 0.7186 | -0.2747 | 0.5528 |
| UJBH | 0.6599 | -0.4516 | 0.3069 |
| UJTDF | 0.9189 | 0.1616 | 0.0666 |
| TLEN | 0.8722 | 0.1738 | -0.2735 |
| UJFLIP | 0.7278 | -0.2936 | -0.08137 |
| UJANU | 0.8266 | 0.06028 | -0.2876 |
| WFLU | 0.5042 | 0.4217 | -0.3266 |
| HD | 0.5073 | 0.673 | -0.2182 |
| LFLIP | -0.2134 | 0.7801 | 0.3331 |
| WFLIP | -0.03503 | 0.4445 | 0.7859 |
| Eigenvalues | 5.1326 | 1.8627 | 1.5279 |
| % Total variance | 46.66 | 16.934 | 13.89 |
| **Cumulative % variance** | **46.66** | **63.59** | **77.484** |
