# Supplementary material for: A New Dolphin Species, the Burrunan Dolphin Tursiops australis sp. nov., Endemic to Southern Australian Coastal Waters
Source: PLoS One. 2011 Sep 14;6(9):e24047. doi: 10.1371/journal.pone.0024047 (PMC3173360; doi:10.1371/journal.pone.0024047)
Supplement: Table S5 — PCR primers used to amplify mtDNA cytochrome b gene and control region from Tursiops maugeanus syntype specimens (DOC) [file pone.0024047.s008.doc]

**Table S5** PCR primers used to amplify mtDNA cytochrome *b* gene and control region from *Tursiops maugeanus* syntype specimens

| **Primer** | **Sequence (5’ > 3’)** | **Amplicon Size (excl. primers)** |
| --- | --- | --- |
| Dolphin_cytb_F1 | ACATCCGAAAAACACACCCACT | 162 |
| Dolphin_cytb_R1 | GGCAGATRTGTGCGACTGAT |  |
| Dolphin_cytb_F2 | TACACRCCAGACACCTCAAC | 187 |
| Dolphin_cytb_R2 | GCCTACGAATGCAGTGGCTAT |  |
| Dolphin_cytb_F3 | TTCCAAGAAACATGAAACATTG | 182 |
| Dolphin_cytb_R3 | AAGTGGAAAGCGAAAAAGCG |  |
| Dolphin_cytb_F4 | TTTCCGTAGATAAAGCAACA | 198 |
| Dolphin_cytb_R4 | GGGGGTGAATAGGGTTARTGC |  |
| Dolphin_cytb_F5 | ACATCCTAGGYGCCTTACTC | 162 |
| Dolphin_cytb_R5 | AATAGCGCTAGGACTCCTCC |  |
| Dolphin_cytb_F6 | ACCCCTGCACACATCAAACC | 200 |
| Dolphin_cytb_R6 | TACGGGTTGGCCTCCAATTC |  |
| Dolphin_cytb_F7 | CCCTTCAGCCAACTCCTATTTTG | 138 |
| Dolphin_cytb_R7 | ACAAGACCGAGGTATTTTGTT |  |
| Dolphin_CR_F1 | ACAGTTACCACAACAYCACA | 147 |
| Dolphin_CR_R1 | ACGAGCTTTAACTTATCGTATGGA |  |
| Dolphin_CR_F2 | ACATGCTATGTATTATTGTGCATTC | 140 |
| Dolphin_CR_R2 | GAGCGGGTTGCTGGTTTCA |  |
| Dolphin_CR_F3 | CTCCATTAGATCACGAGCTT | 132 |
| Dolphin_CR_R3 | ACGAGTGGGCGATTTTAAGT |  |
