# Supplementary material for: A New Dolphin Species, the Burrunan Dolphin Tursiops australis sp. nov., Endemic to Southern Australian Coastal Waters
Source: PLoS One. 2011 Sep 14;6(9):e24047. doi: 10.1371/journal.pone.0024047 (PMC3173360; doi:10.1371/journal.pone.0024047)
Supplement: Table S6 — GenBank accession numbers and species information for samples incorporated to phylogenetic analyses (DOC) [file pone.0024047.s009.doc]

**Table S6** GenBank accession numbers and species information for samples incorporated to phylogenetic analyses

| **Genbank Accession** | **Haplotype code** | **Species** | **Reference** |
| --- | --- | --- | --- |
| GQ504119 | Dd88 | *Delphinus delphis* | Kingston *et al* 2009 |
| GQ504116 | Dd169 | *Delphinus delphis* | Kingston *et al* 2009 |
| GQ504117 | Dd170 | *Delphinus delphis* | Kingston *et al* 2009 |
| GQ504151 | Dd209 | *Delphinus delphis* | Kingston *et al* 2009 |
| EU12120 | PR842 | *Lagenodelphis hosei* | Caballero *et al* 2008 |
| EU12119 | PR571 | *Lagenodelphis hosei* | Caballero *et al* 2008 |
| DQ845438 | Lhos001 | *Lagenodelphis hosei* | Kingston *et al* 2009 |
| DQ845439 | Lhos003 | *Lagenodelphis hosei* | Kingston *et al* 2009 |
| AF113487 | Lacu | *Lagenorhynchus acutus* | Cipriano 1997 |
| EF027091 | SfluCC | *Sotalia fluviatilis* | Caballero *et al* 2007 |
| EF027087 | SfluX | *Sotalia fluviatilis* | Caballero *et al* 2007 |
| EF027089 | SfluY | *Sotalia fluviatilis* | Caballero *et al* 2007 |
| EF027067 | SguiE | *Sotalia guianensis* | Caballero *et al* 2007 |
| EF027079 | SguiR | *Sotalia guianensis* | Caballero *et al* 2007 |
| EF027073 | SguiL | *Sotalia guianensis* | Caballero *et al* 2007 |
| GQ504132 | Schi1 | *Sousa chinensis* | Kingston *et al* 2009 |
| GQ504135 | Schi5 | *Sousa chinensis* | Kingston *et al* 2009 |
| GQ504133 | Schi2 | *Sousa chinensis* | Kingston *et al* 2009 |
| GQ504136 | Schi6 | *Sousa chinensis* | Kingston *et al* 2009 |
| DQ845442 | SA1 | *Stenella attenuata* | Kingston *et al* 2009 |
| GQ504121 | SA3 | *Stenella attenuata* | Kingston *et al* 2009 |
| DQ845443 | SA5 | *Stenella attenuata* | Kingston *et al* 2009 |
| GQ504123 | SA6 | *Stenella attenuata* | Kingston *et al* 2009 |
| GQ504130 | SA13 | *Stenella attenuata* | Kingston *et al* 2009 |
| DQ845447 | Scl12 | *Stenella clymene* | Kingston *et al* 2009 |
| GQ504146 | SCL6 | *Stenella clymene* | Kingston *et al* 2009 |
| GQ504141 | SCL14 | *Stenella clymene* | Kingston *et al* 2009 |
| GQ504176 | SF15 | *Stenella frontalis* | Kingston *et al* 2009 |
| GQ504192 | SF38 | *Stenella frontalis* | Kingston *et al* 2009 |
| GQ504193 | SF39 | *Stenella frontalis* | Kingston *et al* 2009 |
| GQ504194 | SF40 | *Stenella frontalis* | Kingston *et al* 2009 |
| GQ504195 | SF47 | *Stenella frontalis* | Kingston *et al* 2009 |
| DQ845445 | SL1 | *Stenella longirostris* | Kingston *et al* 2009 |
| GQ504169 | SL7 | *Stenella longirostris* | Kingston *et al* 2009 |
| GQ504166 | SL3 | *Stenella longirostris* | Kingston *et al* 2009 |
| HQ115064 | SEAust6 | *Tursiops aduncus* | Wiszniewski *et al* 2010 |
| GQ420670 | SEAust8 | *Tursiops aduncus* | Wiszniewski *et al* 2010 |
| AF056243 | XM-95-07 | *Tursiops aduncus* | Wang *et al* 1999 |
| AF056241 | PE-94-13 | *Tursiops aduncus* | Wang *et al* 1999 |
| AF049100 | PE-05 | *Tursiops aduncus* | Wang *et al* 1999 |
| AF287952 | SEAust2 | *Tursiops aduncus* | Moller and Beheregaray 2001 |
| AF287954 | SEAust4 | *Tursiops aduncus* | Moller and Beheregaray 2001 |
| GQ504114 | Tadu1 | *Tursiops aduncus* | Kingston *et al* 2009 |
| AF459520 | Tadu32 | *Tursiops aduncus* | Ji *et al* unpublished |
| AF459518 | Tadu0398 | *Tursiops aduncus* | Ji *et al* unpublished |
| DQ517442 | Tadu holo | *Tursiops aduncus* | Perrin *et al* 2007 |
| EF192140 | KB01 | *Tursiops* sp. | Bilgmann *et al* 2007 |
| EF192141 | KB02 | *Tursiops* sp. | Bilgmann *et al* 2007 |
| EF192142 | KB03 | *Tursiops* sp. | Bilgmann *et al* 2007 |
| EF192144 | KB05 | *Tursiops* sp. | Bilgmann *et al* 2007 |
| EF192145 | KB06 | *Tursiops* sp. | Bilgmann *et al* 2007 |
| EF192146 | KB07 | *Tursiops* sp. | Bilgmann *et al* 2007 |
| EF192147 | KB08 | *Tursiops* sp. | Bilgmann *et al* 2007 |
| EF192148 | KB09 | *Tursiops* sp. | Bilgmann *et al* 2007 |
| EF192149 | KB10 | *Tursiops* sp. | Bilgmann *et al* 2007 |
| EU276411 | NZ-F10 | *Tursiops truncatus* | Tezanos-Pinto *et al* 2009 |
| EU276409 | NZ-MS25 | *Tursiops truncatus* | Tezanos-Pinto *et al* 2009 |
| EU276398 | NZ-N35 | *Tursiops truncatus* | Tezanos-Pinto *et al* 2009 |
| EU276392 | NZ-N06 | *Tursiops truncatus* | Tezanos-Pinto *et al* 2009 |
| EU276390 | NZ-N18 | *Tursiops truncatus* | Tezanos-Pinto *et al* 2009 |
| DQ845448 | Tt001 | *Tursiops truncatus* | Kingston *et al* 2009 |
