# Supplementary material for: A New Dolphin Species, the Burrunan Dolphin Tursiops australis sp. nov., Endemic to Southern Australian Coastal Waters
Source: PLoS One. 2011 Sep 14;6(9):e24047. doi: 10.1371/journal.pone.0024047 (PMC3173360; doi:10.1371/journal.pone.0024047)
Supplement: Table S7 — Discriminant function analysis loadings on 34 cranial measures from 40 ‘bottlenose’ dolphin skulls (DOC) [file pone.0024047.s010.doc]

**Table S7 Canonical Discriminant Functions loadings on 33 cranial measures from 40 ‘bottlenose’ dolphin skulls**

| **Cranial Character** | **Factor 1** | **Factor 2** |
| --- | --- | --- |
| **APAP** | -1.54 | -1.545 |
| **CBL** | 0.238 | 2.011 |
| **DFWM** | -2.254 | -0.341 |
| **DFWN** | -0.325 | 0.048 |
| **GLPT** | 1.004 | 1.42 |
| **GLPTF** | -1.092 | -1.1 |
| **GPOW** | 8.64 | -4.373 |
| **GPRW** | -0.339 | 4.109 |
| **GWEN** | -2.629 | 0.561 |
| **GWIN** | -0.193 | -0.974 |
| **GWPTF** | 0.111 | 0.728 |
| **GWPX** | 3.602 | -1.281 |
| **LAL** | -2.353 | -1.386 |
| **LO** | -1.13 | 0.629 |
| **LTRL** | -1.992 | 0.49 |
| **LWPTF** | -0.645 | -0.688 |
| **MFL** | 0.884 | -1.008 |
| **MH** | -3.99 | 1.514 |
| **ML** | 5.714 | -0.628 |
| **MSL** | 1.814 | -1.12 |
| **PRW** | -1.673 | 0.967 |
| **RL** | -7.5 | 0.221 |
| **RW60** | 7.647 | 5.909 |
| **RW75** | 2.869 | 0.56 |
| **RWB** | -2.85 | -1.721 |
| **RWM** | -5.606 | -5.662 |
| **TPC** | 0.987 | 1.291 |
| **TREN** | -3.678 | -0.365 |
| **TRIN** | 7.856 | -4.968 |
| **UTLTR** | 1.347 | -0.001 |
| **VW** | -0.979 | -0.827 |
| **WAS** | -0.619 | -0.497 |
| **ZW** | -2.595 | 0.614 |
