# Supplementary material for: A New Dolphin Species, the Burrunan Dolphin Tursiops australis sp. nov., Endemic to Southern Australian Coastal Waters
Source: PLoS One. 2011 Sep 14;6(9):e24047. doi: 10.1371/journal.pone.0024047 (PMC3173360; doi:10.1371/journal.pone.0024047)
Supplement: Table S8 — Discriminant function analysis loadings for 11 external measures from 17 ‘bottlenose’ dolphins (DOC) [file pone.0024047.s011.doc]

**Table S8** Discriminant function analysis loadings for 11 external measures from 17 'bottlenose' dolphins

| **External measure** | **DFA loading** |
| --- | --- |
| TLEN | 1.4744 |
| UJGAP | -4.686 |
| UJTDF | -0.43225 |
| UJFLIP | -0.214 |
| UJANU | -2.9317 |
| UJEYE | 4.8313 |
| UJBH | -6.5718 |
| WFLU | -3.0279 |
| HD | 2.9126 |
| LFLIP | -2.4657 |
| WFLIP | 0.87623 |
