# Supplementary material for: A New Dolphin Species, the Burrunan Dolphin Tursiops australis sp. nov., Endemic to Southern Australian Coastal Waters
Source: PLoS One. 2011 Sep 14;6(9):e24047. doi: 10.1371/journal.pone.0024047 (PMC3173360; doi:10.1371/journal.pone.0024047)
Supplement: Table S9 — Species classification overview based on different analyses and characters (DOC) [file pone.0024047.s012.doc]

**Table S9** Species classification overview based on different analyses and characters.

|  |  | **Classification** | |  |  |  |
| --- | --- | --- | --- | --- | --- | --- |
| **Cranial** | **Ext. morph.** | Cluster analysis 'group' | | mtDNA type | CR | cyt b |
| **code** | **code** | Cranial | Ext. morph. |  | haplotype | haplotype |
| C29579 | - | 1 |  |  |  |  |
| C29587 | - | 1 |  |  |  |  |
| C29667 | - | 1 |  |  |  |  |
| C24944 | - | 1 |  |  |  |  |
| C28760 | - | 1 |  |  |  |  |
| C29580 | - | 1 |  |  |  |  |
| C29577 | - | 1 |  | *Tursiops australis* | Burru CR2 |  |
| C29586 | - | 1 |  | *Tursiops australis* | Burru CR2 |  |
| C10357 | - | 1 |  | *Tursiops australis* | Burru CR2 |  |
| C31642 | - | 1 |  |  |  |  |
| C35986 | - | 1 |  | *Tursiops australis* | Burru CR6 | Burru Cytb1 |
| C35987 | - | 1 |  | *Tursiops australis* | Burru CR6 |  |
| C25071 | - | 1 |  |  |  |  |
| Unknown | - | 1 |  |  |  |  |
| C29506 | - | 1 |  |  |  |  |
| C11271 | - | 1 |  |  |  |  |
| **1365*** | **-** | **1** |  | ***Tursiops australis*** | **Burru CR6** | **Burru Cytb1** |
| A1759 | - | 1 |  | *Tursiops australis* | Burru CR6 |  |
| A2430 | - | 1 |  |  |  |  |
| 1946/7 | - | 1 |  |  |  |  |
| 1972/1/35 | - | 2 |  |  |  |  |
| 1360** | - | 2 |  | *Tursiops truncatus* | CRTT29 | TT Cytb5 |
| C31643 | - | 2 |  |  |  |  |
| A2425 | - | 2 |  |  |  |  |
| A198 | - | 2 |  |  |  |  |
| C24987 | - | 2 |  |  |  |  |
| C29585 | - | 2 |  |  |  |  |
| C29581 | - | 2 |  |  |  |  |
| TMAG unreg | - | 2 |  |  |  |  |
| WAPSTRA | - | 2 |  |  |  |  |
| C35965 | MU141206a | 1 | 1 | *Tursiops australis* | Burru CR6 |  |
| C35985 | MU011206 | 1 | 1 | *Tursiops australis* | Burru CR6 | Burru Cytb1 |
| C35966 | MU141206b | 1 | 1 | *Tursiops australis* | Burru CR8 | Burru Cytb1 |
| C36750 | MU041107 | 1 | 1 | *Tursiops australis* | Burru CR6 | Burru Cytb1 |
| C35969 | MU080306 | 2 | 2 | *Tursiops truncatus* | CRTT14 | TT Cytb12 |
| C35968 | MU251007 | 1 | 1 | *Tursiops australis* | Burru CR6 | Burru Cytb1 |
| MU210108 | MU210108 | 1 | 1 | *Tursiops australis* | Burru CR2 |  |
| MU230108 | MU230108 | 1 | 1 | *Tursiops australis* | Burru CR2 | Burru Cytb3 |
| MU230607 | MU230607 | 2 | 2 | *Tursiops truncatus* | CRTT28 | TT Cytb12 |
| MU220108 | MU220108 | 2 | 2 | *Tursiops truncatus* | CRTT1 | TT Cytb14 |
| - | MU021108 | - | 1 | *Tursiops australis* | Burru CR8 | Burru Cytb1 |
| - | MU291007 | - | 1 | *Tursiops australis* | Burru CR8 | Burru Cytb1 |
| - | MU230407 | - | 1 | *Tursiops australis* | Burru CR2 | Burru Cytb4 |
| - | MU190905 | - | 1 | *Tursiops australis* | Burru CR2 | Burru Cytb4 |
| - | MU271006 | - | 1 | *Tursiops australis* | Burru CR2 | Burru Cytb3 |
| - | MU280405 | - | 2 | *Tursiops truncatus* | CRTT2 | TT Cytb5 |
| - | MU010709 | - | 2 | *Tursiops truncatus* | CRTT2 | TT Cytb5 |
| JM1230 | - | 3 |  |  |  |  |
| JM11375 | - | 3 |  |  |  |  |
| 5241 | - | 3 |  |  |  |  |
| 6428 | - | 3 |  |  |  |  |
| 4155 | - | 3 |  |  |  |  |

**Group 1** *Tursiops australis* sp. nov.; **Group 2** *Tursiops truncatus*; **Group 3** *Tursiops aduncus*; ******Tursiops australis* sp. nov. holotype; *******Tursiops maugeanus* lectotype; **CR** mtDNA control region; **Cytb** mtDNA cytochrome *b* region; **Burru** *Tursiops australis* sp. nov.; **TT** *Tursiops truncatus.*
