# Supplementary material for: A New Dolphin Species, the Burrunan Dolphin Tursiops australis sp. nov., Endemic to Southern Australian Coastal Waters
Source: PLoS One. 2011 Sep 14;6(9):e24047. doi: 10.1371/journal.pone.0024047 (PMC3173360; doi:10.1371/journal.pone.0024047)
Supplement: Table S10 — GenBank accession numbers of the sequences from this study (DOC) [file pone.0024047.s013.doc]

**Table S10** GenBank accession numbers of the sequences from this study

| **GenBank accession no.** | **Haplotype code** | **Species** | **mtDNA region** |
| --- | --- | --- | --- |
| JN571481 | BurruCR6 | *Tursiops australis* holotype | control region |
| JN571464 | BurruCR2 | *Tursiops australis* | control region |
| JN571465 | BurruCR6 | *Tursiops australis* | control region |
| JN571466 | BurruCR8 | *Tursiops australis* | control region |
| JN571467 | BurruCR1* | *Tursiops australis* | control region |
| JN571468 | BurruCR3* | *Tursiops australis* | control region |
| JN571469 | BurruCR7* | *Tursiops australis* | control region |
| JN571470 | CRTT29 | *Tursiops truncatus* | control region |
| JN571471 | CRTT1 | *Tursiops truncatus* | control region |
| JN571472 | CRTT2 | *Tursiops truncatus* | control region |
| JN571473 | CRTT14 | *Tursiops truncatus* | control region |
| JN571474 | CRTT28 | *Tursiops truncatus* | control region |
| JN571482 | BurruCytb1 | *Tursiops australis* holotype | cytochrome *b* gene |
| JN571475 | BurruCytb1 | *Tursiops australis* | cytochrome *b* gene |
| JN571476 | BurruCytb3 | *Tursiops australis* | cytochrome *b* gene |
| JN571477 | BurruCytb4 | *Tursiops australis* | cytochrome *b* gene |
| JN571478 | TTCytb5 | *Tursiops truncatus* | cytochrome *b* gene |
| JN571479 | TTCytb12 | *Tursiops truncatus* | cytochrome *b* gene |
| JN571480 | TTCytb14 | *Tursiops truncatus* | cytochrome *b* gene |
