# Supplementary material for: A New Dolphin Species, the Burrunan Dolphin Tursiops australis sp. nov., Endemic to Southern Australian Coastal Waters
Source: PLoS One. 2011 Sep 14;6(9):e24047. doi: 10.1371/journal.pone.0024047 (PMC3173360; doi:10.1371/journal.pone.0024047)
Supplement: Table S11 — Average cranial measures (mm) and tooth counts for Tursiops australis, Tursiops truncatus, Tursiops aduncus (current study) and from Tursiops aduncus holotype (Perrin et al. 2007) and Tursiops adunucs (Wang et al. 2000) (DOC) [file pone.0024047.s014.doc]

**Table S11** Average cranial measures (mm) and tooth counts for *Tursiops australis*, *Tursiops truncatus*, *Tursiops aduncus* (current study) and from *Tursiops aduncus* holotype (Perrin et al. 2007) and *Tursiops adunucs* (Wang et al. 2000)

|  | ***Tursiops australis*** |  | ***Tursiops truncatus*** |  | ***Tursiops aduncus*** |  | ***Tursiops aduncus* holotype** |  | ***Tursiops aduncus*** (Wang et al 2000) | | |
| --- | --- | --- | --- | --- | --- | --- | --- | --- | --- | --- | --- |
|  | current study |  | current study |  | current study |  | (Perrin et al 2007) |  | Chinese waters |  | South African waters |
| **Measure** | Mean (mm) |  | Mean (mm) |  | Mean (mm) |  | Mean (mm) |  | Mean (mm) |  | Mean (mm) |
| CBL | 493.58 |  | 527.88 |  | 441 |  | 479 |  | 485.1 |  | 472.7 |
| GLPTF | 114.59 |  | 115.85 |  | 95.69 |  | 108 |  | - |  | - |
| GWPTF | 82.96 |  | 84.28 |  | 76.86 |  | 74 |  | - |  | - |
| GWEN | 60.42 |  | 58.95 |  | 50.05 |  | 54 |  | 58.7 |  | 54.4 |
| GWIN | 66.98 |  | 76.64 |  | 57.59 |  | 64 |  | - |  | - |
| GPRW | 213.35 |  | 237.62 |  | 193.90 |  | 194 |  | 201.9 |  | 203.4 |
| GPOW | 238.93 |  | 268.58 |  | 213.00 |  | 234 |  | 223.4 |  | 230.2 |
| GWPX | 94.72 |  | 97.98 |  | 77.46 |  | 84 |  | 86.2 |  | 83.4 |
| GPARW | 185.45 |  | 190.16 |  | - |  | 166 |  | - |  | - |
| LAL | 52.03 |  | 62.23 |  | 42.68 |  | 42 |  | 46.1 |  | 44.8 |
| LO | 69.95 |  | 69.06 |  | 60.63 |  | 62 |  | - |  | - |
| LTRL | 232.87 |  | 247.83 |  | 219.90 |  | - |  | 243.8 |  | 226.9 |
| MH | 91.78 |  | 97.48 |  | 80.25 |  | - |  | 82.6 |  | 83.2 |
| ML | 423.30 |  | 457.46 |  | 373.20 |  | - |  | 415 |  | 399.6 |
| PRW | 48.49 |  | 50.20 |  | 33.31 |  | 31 |  | - |  | - |
| RL | 280.37 |  | 303.69 |  | 254.30 |  | 276 |  | 282 |  | 271.9 |
| RWB | 132.58 |  | 143.05 |  | 103.38 |  | 117 |  | 115.8 |  | 112.3 |
| RWM | 79.44 |  | 88.84 |  | 62.64 |  | 60 |  | 64.2 |  | 64.9 |
| RW75 | 63.48 |  | 70.97 |  | 50.05 |  | 46 |  | 50.3 |  | 48.8 |
| TREN | 327.98 |  | 353.23 |  | 295.20 |  | 316 |  | 328.5 |  | 316.9 |
| TRIN | 333.43 |  | 360.58 |  | 297.17 |  | 327 |  | - |  | - |
| UTLTR | 236.59 |  | 253.04 |  | 209.10 |  | 231 |  | 236.9 |  | 224.8 |
| WAS | 161.01 |  | 176.74 |  | 159.02 |  | 30 |  | - |  | - |
| TPC | 73.41 |  | 85.48 |  | 62.42 |  | 199 |  | - |  | - |
| ZW | 228.52 |  | 263.27 |  | 204.50 |  | 232 |  | 230.6 |  | 229.6 |
| **Tooth counts** |  |  |  |  |  |  |  |  |  |  |  |
| TTLL | 22.84 |  | 22.09 |  | 24.20 |  | 26 |  | 25.7 |  | 25.9 |
| TTLR | 23.12 |  | 22.18 |  | 24.20 |  | 28 |  | 25.6 |  | 26.1 |
| TTUL | 23.88 |  | 23.36 |  | 23.20 |  | 27 |  | 2.2 |  | 25.8 |
| TTUR | 23.85 |  | 23.27 |  | 23.00 |  | 27 |  | 25.4 |  | 25.3 |
